# Novel 3-chloro-6-nitro-1*H*-indazole derivatives as promising antileishmanial candidates: synthesis, biological activity, and molecular modelling studies

**DOI:** 10.1080/14756366.2021.1995380

**Published:** 2021-12-11

**Authors:** Mohamed Mokhtar Mohamed Abdelahi, Youness El Bakri, Chin-Hung Lai, Karthikeyan Subramani, El Hassane Anouar, Sajjad Ahmad, Mohammed Benchidmi, Joel T. Mague, Jelena Popović-Djordjević, Souraya Goumri-Said

**Affiliations:** a Laboratoire de Chimie Organique Hétérocyclique, Centre de Recherche des Sciences des Médicaments, Pôle de Compétences Pharmacochimie, URAC 21, Faculté des Sciences, Mohammed V University Rabat, Rabat, Morocco; b Department of Theoretical and Applied Chemistry, South Ural State University, Chelyabinsk, Russia; c Department of Medical Applied Chemistry, Chung Shan Medical University, Taichung, Taiwan; d Department of Medical Education, Chung Shan Medical University Hospital, Taichung, Taiwan; e G.S. Gill Research Institute, Guru Nanak College, Chennai, India; f Department of Chemistry, College of Sciences and Humanities in Al-Kharj, Prince Sattam Bin Abdulaziz University, Al-Kharj, Saudi Arabia; g Department of Health and Biological Sciences, Abasyn University, Peshawar, Pakistan; h Department of Chemistry, Tulane University, New Orleans, LA, USA; i Department for Chemistry and Biochemistry, Faculty of Agriculture, University of Belgrade, Belgrade, Serbia; j College of Science, Physics Department, Alfaisal University, Riyadh, Saudi Arabia

**Keywords:** 1,2,3-Triazole, isooxazoline, isoxazole, antileishmanial activity, molecular dynamics

## Abstract

An efficient pathway was disclosed for the synthesis of 3-chloro-6-nitro-1*H*-indazole derivatives by 1,3-dipolar cycloaddition on dipolarophile compounds **2** and **3**. Faced the problem of separation of two regioisomers, a click chemistry method has allowed us to obtain regioisomers of triazole-1,4 with good yields from 82 to 90% were employed. Also, the antileishmanial biological potency of the compounds was achieved using an MTT assay that reported compound **13** as a promising growth inhibitor of *Leishmania major*. Molecular docking demonstrated highly stable binding with the Leishmania trypanothione reductase enzyme and produced a network of hydrophobic and hydrophilic interactions. Molecular dynamics simulations were performed for TryR-**13** complex to understand its structural and intermolecular affinity stability in a biological environment. The studied complex remained in good equilibrium with a structure deviation of ∼1–3 Å. MM/GBSA binding free energies illustrated the high stability of TryR-**13** complex. The studied compounds are promising leads for structural optimisation to enhance the antileishmanial activity.

## 1. Introduction

Cutaneous leishmaniasis (CL) is an extremely polymorphic and heterogeneous group of diseases. A prevalent feature is that they are caused by parasites of the genus Leishmania transmitted by the bite of midges belonging to the subfamily Phebotomidae and commonly known as sand flies^1^. This disease is associated with high morbidity and mortality rates and currently affects more than 12 million people worldwide in 88 countries, mostly in equatorial and subtropical areas^2^^,^^3^. In the Old World (Europe, Africa, Central Asia, and Middle East), the ulcerated skin lesions typical for CL are mainly triggered by *Leishmania major* and *Leishmania tropica*. Meanwhile, in the New World (Latin America), they are induced by *Leishmania braziliensis*, *Leishmania guyanensis*, and *Leishmania mexicana* species complexes, of which the former two species complexes can disseminate to the nasopharyngeal tissues and cause eradicated mucosal forms (mucocutaneous leishmaniasis)^4^. Besides CL, visceral leishmaniasis (VL), also termed as kala-azar is severe Leishmaniasis and is associated with high mortality if left untreated^5^. VL is cause by L. infantum and often prevalent in Mediterranean and Latin America^6^. The pathogen is an unusual cause of CL^7^. The pathways and the mechanisms that lead to inhibition or induction of apoptosis in Leishmania spp. are of particular interest as they will be possible targets for the development of antileishmania drugs^5^.

The study of heterocycles is a branch of organic chemistry that attracts much attention from chemists working not only in the area of natural products but also in synthetic chemistry^6–33^. Moreover, many useful drugs have emerged from the successful investigation carried out in this area. The indazole nucleus is a very important heterocyclic framework in medicinal chemistry. This scaffold is present in a large number of compounds with a wide range of pharmacological activities which include anticancer^6^, antimalarial^7^, anti-tubercular^8^, antimicrobial^9^, and antidiabetic function^10^.

Indazoles correspond to isomeric chemical compounds with molecular formula C_7_H_6_N_2_, having a pyrazole ring condensed with a benzene ring (benzo[*c*]pyrazole, 1,2-benzodiazole). The indazole heterocycle is normally referred to as 1*H*-indazole, although it has two other potential tautomers 2*H*-indazole and 3*H*-indazole^34^. Isoxazolines are an important class of nitrogen and oxygen-containing heterocycles that belong to the azoles family which have much importance in the field of medicinal chemistry. Isoxazoles are considered privileged scaffolds in drug discovery and have a broad spectrum of activities, such as antiviral^14^, antibacterial^15^, antimycobacterial^16^, anti‐inflammatory^17^, and more recently, they have also demonstrated antitrypanosomal activity^18^.

1,2,3-triazole, obtained by highly versatile, efficacious, and selective “Click Reaction” has become a synthetic/medicinal chemist’s favourite not only because of its ability to mimic different functional groups but also due to enhancement in the targeted biological activities. Triazole ring has also been shown to play a critical role in biomolecular mimetics, fragment-based drug design, and bio-orthogonal methodologies. 1,2,3-triazole derivatives possess significant biological and pharmacological properties, inclusive of anti-Alzheimer’s disease^19^^,^^20^, anticancer^21^^,^^22^, antimalarial^23^^,^^24^, antitubercular^25^^,^^26^, antiviral^27^^,^^28^, and antibacterial activity^29^. Therefore, 1,2,3-triazole derivatives are privileged scaffolds for the development of novel drugs^35–38^.

As part of our research on the synthesis of recent heterocycles capable of exhibiting biological and pharmacological activities, the use of 1,3-dipolar cycloaddition reactions in the synthesis of the new heterocyclic systems containing two heterocycles separated by a methylene group comprising 6-nitroindazole and either isoxazoline, 1,2,3-triazole or isoxazole have been investigated. In addition, synthesised compounds have been tested for antileishmanial activity and explored by computational docking and molecular dynamics simulation studies to predict their binding conformation and stable binding with Leishmania trypanothione reductase (TryR) enzyme^39^.

## 2. Experimental and calculated methods

### 2.1. Chemicals

All reagents were purchased from commercial suppliers and were employed without further purification. The reactions were monitored by thin-layer chromatography (TLC) analysis using silica gel (60 F254) plates. Flash column chromatography was performed on silica gel 60 (230–400 mesh, 0.040–0.063 mm). Melting points (in °C) were taken on samples in open capillary tubes and are uncorrected. ^1^H NMR spectra (300 MHz) and ^13 ^C NMR spectra (75 MHz) were recorded on a Varian Gemini spectrometer with tetramethylsilane (TMS) as the internal reference. Mass spectra were performed on a Perkin-Elmer SCIEX API unit 300. The samples were ionised by the electrospray technique (ESI). Chemical shifts are given in parts per million from tetramethylsilane (TMS) as internal standard. The following abbreviations are used for the proton spectra multiplicities: s: singlet, d: doublet, t: triplet, q: quartette, qt: quartplet, m: multiplet. Coupling constants (J) are reported in Hertz (Hz).

### 2.2. Parasites culture

The *in vitro* antileishmanial effect of the compounds was evaluated on the culture of three Leishmania species: *Leishmania infantum* (MHOM/MA/1998/LVTA), *Leishmania tropica* (MHOM/MA/2010/LCTIOK-4), and *Leishmania major* (MHOM/MA/2009/LCER19-09). The promastigote forms were isolated and identified in the National Reference Laboratory of Leishmaniasis, National Institute of Health, Rabat, Morocco.

### 2.3. Cell viability assays

Parasite cultures of each Leishmania species were washed with phosphate-buffered saline (PBS) and centrifuged at 1500 rpm for 10 min. Cells were then re-suspended in RPMI 1640 (GIBCO) supplemented with 10% heat-inactivated foetal calf serum and 1% of Penicillin-Streptomycin mixture. Cultures were maintained at 23 °C. The effect of compounds on cell viability was assessed using the 3-(4.5-dimethylthiazol-2-yl)-2.5-diphenyl-tetrazolium bromide (MTT) assay. MTT assays are presently the preferred method of cytotoxicity assessment in our laboratory^14^. The tests were conducted on 96-well plates. Before treatment with extracts, 100 μl medium RPMI (GIBCO) containing 2.5 × 10^6^ promastigotes/mL were placed in each well-containing RPMI (GIBCO) and cultured at 23 °C for 72 h. After incubation, samples were treated with compounds. Exactly from the stock solution (10 mg/mL), each extracted sample was applied in a series of six dilutions (final concentrations ranging to 200 μg/mL) in dimethyl sulfoxide (DMSO 1%). A test solution (100 μL) was added in decreasing concentrations in triplicate. The microplate was then incubated for 72 h at 23 °C. After that, 10 μL of MTT solution (5 mg/mL) (SIGMA) were added to the wells containing the samples and were incubated for 3 h at 23 °C. Tetrazolium salts are cleaved to formazan dye by cellular enzyme mitochondrial succinate dehydrogenase (only in the viable promastigotes). A solubilisation solution (isopropanol/hydrochloric acid) was added to dissolve the insoluble purple formazan product into colouration solution. The absorbance was measured at 570 nm, using a microplate reader (Statfax 2100). Data are presented as means ± *SD* of three different assays. Statistical analysis was performed by using “Origin 9.0” software.

### 2.4. Molecular docking details

The binding modes of the active 3-chloro-6-nitro-1*H*-indazole derivatives (**4**, **5**, **11**, and **13**) as potent antileishmanial agents against *Leishmania infantum* trypanothione reductase (TryR) were predicted using the Autodock 4.0 packages^40^. TryR was selected as a receptor in docking studies based on the literature reported works where this enzyme was targeted by indazole derivatives^31–33^. X-ray coordinates of TryR (PDB codes 2JK6) and its corresponding co-crystallized docked ligand flavin adenine dinucleotide were retrieved from RCSB Protein Data Bank (PDB). As a starting step, water molecules were removed, polar hydrogen atoms and Kollman charges were added to the extracted receptor structure using AutoDock Tools. The active site information was extracted from the enzyme crystal structure. Re-docking of the original ligand flavin adenine dinucleotide into the active site of trypanothione reductase was conducted to validate docking protocol and is well reproduced with RMSD values of 1.16 Å. 3D molecular structures geometries of 3-chloro-6-nitro-1*H*-indazole derivatives (**4**, **5**, **11**, and **13**) were minimised via the Merck molecular force field 94 (MMFF94). The optimised geometries were saved as pdb files. Non-polar hydrogens were merged and rotatable bonds were defined for each docked ligand. The docking study was performed following the same steps used in our previous methodology^41^.

### 2.5. Molecular dynamics details

The top-ranked receptor-ligand complex was generated and subjected to molecular simulation protocol using Desmond. The protein system was built involving periodic boundary conditions with a 10 Å^3^ orthorhombic box from the centre of mass of the protein. TIP3P water solvation system was used as a buffer system with charged ions placed isotropically to neutralise the Ewald charge summation of the solvated protein entity. The system was minimised with maximum iterations of 5000 steps with a gradient convergence threshold of 1.0 kcal mol^−1 ^Å^−1^. Once the system is minimised, the system was subjected to Newtonian dynamics of the model system to evaluate the energy of the system. Two ps steps were integrated to record the simulation. A six-stage NPT ensemble default relaxation process was carried out before performing molecular dynamics simulations. Initially, at the first stage, solute restrained Brownian dynamics of the ensemble was carried out by keeping the energy constant using the NVT condition. In the second stage using a Berendsen thermostat the NVT (canonical) ensemble was allowed to relax with respect to temperature with velocity resampling of every 1 ps applied to the non-hydrogen solute sample. Subsequently, the NVT ensemble was changed to an NPT ensemble with a Berendsen barostat with the system was kept at 1 atm pressure followed by a system equilibration of 1 ns. Then the ensemble was subjected to 50 ns molecular dynamics run. The different post-simulation investigation was performed on the simulated trajectories to test stable binding and binding conformation of the compounds to the receptor TryR. These assessments include root mean square deviation (RMSD), root mean square fluctuations (RMSF), and hydrogen bonds analysis. The MM/GBSA binding free energies of compound 13-TryR complex were carried out using MMPBSA.py module of AMBER20 exploiting 100 frames collected at equal intervals from the simulation trajectories.

### 2.6. Density functional theory computational details

The structures in the gas phase of compounds **4–15** were optimised employing density functional theory (DFT). The DFT calculation was performed by the hybrid B3LYP method which is based on the idea of Becke and considers a mixture of the exact Hartree-Fock (HF) and DFT exchange utilising the B3 functional, together with the LYP correlation functional^42^^,^^43^. In conjunction with the def2-SVP basis set, the B3LYP calculation was carried out^44^. After obtaining the converged geometry, the harmonic vibrational frequencies were calculated at the same theoretical level to confirm that the number of imaginary frequencies is zero for the stationary point. All the calculations in this study were done by the Gaussian 16 program^45^.

## 3. Results and discussion

### 3.1. Synthesis of dipolarophiles

**Scheme 1.**
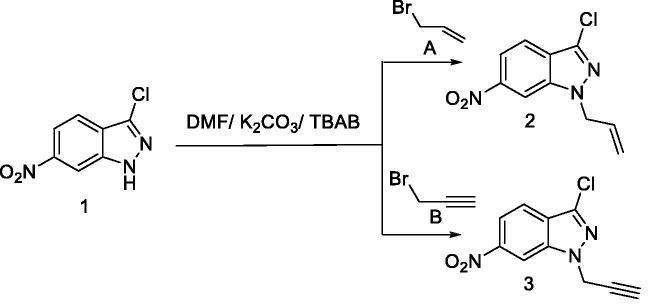
Synthesis of dipolarophiles **2** and **3**.

Dipolarophiles **2** and **3** have been prepared in good yields (88–92%) via alkylation reactions of **1** with allyl bromide or propargyl bromide under phase transfer catalysis conditions using tetra-*n*-butylammonium bromide (TBAB) as catalyst and potassium carbonate as a base in dimethylformamide at room temperature (Scheme 1).

The structures of compounds isolated have been identified by ^1^H NMR and ^13 ^C NMR spectral data. The ^1^H NMR spectrum (DMSO-d6) of **2** shows an allylic proton signal at 5.23 ppm and a multiplet centred at 6.04 (6.14, 6.03, and 6.01) ppm attributable to vinyl protons CH = CH_2_. The ^13 ^C NMR spectra of **2** show the presence of a characteristic signal of the allyl group at 133.92 and one at 115.45 ppm corresponding to the vinylic CH and the methylene vinyl group. Two signals correlating to the methylene group adjacent to the nitrogen atoms appear at 51.63 ppm.

For **3**, the ^1^H NMR spectrum in DMSO-d6 illustrates signals for the propargyl group as a doublet at 4.74, 4.90, and 4.86 ppm, respectively, and a triplet centred at 2.20 (2.21) and 3.31 ppm corresponding to methylene groups bonded to the nitrogen atom and acetylenic HC≡C-proton, respectively. The ^13 ^C NMR spectra show the signal of the terminal acetylenic carbon at 75.0, 75.5, and 75.47 ppm, respectively.

### 3.2. Synthesis of bi-heterocycles containing isoxazoline

**Scheme 2.**
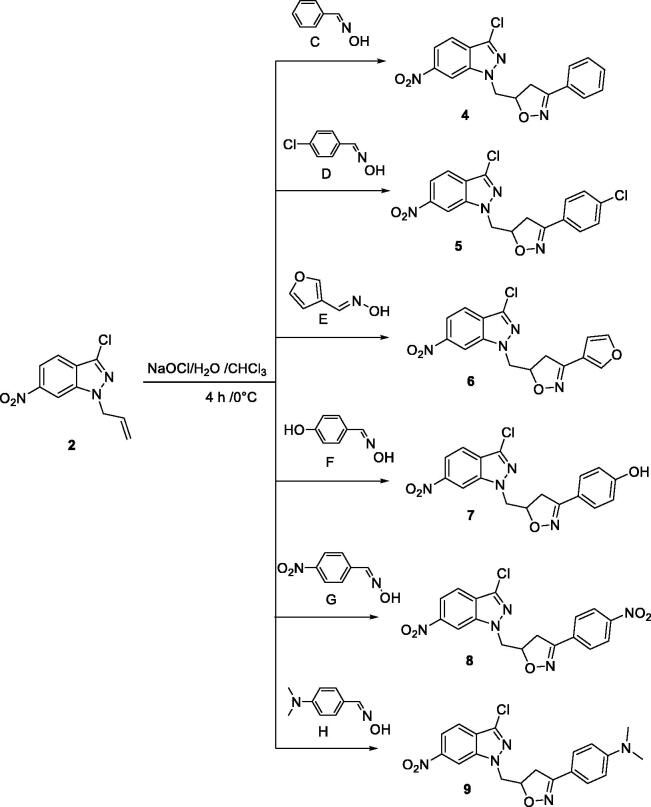
Synthesis of new isoxazoline-6-nitro-1*H*-indazole derivatives.

In Scheme 2, the reaction of the dipolarophile **2** with the dipoles (**C**, **D**, **E**, **F**, **M**, and **P**) in a biphasic medium (bleach/chloroform) at temperatures between −5 and 0 °C, for 4 h with the arylnitriloxides generated *in situ*, leads to the cycloadducts **4–9** with a good yield.

The reaction appears to be region-specific since the heteroatom of the dipole attacks the least hydrogenated carbon of the allyl group in agreement with those already described in the literature^46^. The structures of the heterocyclic products were determined by the ^1^H NMR and ^13 ^C NMR spectral data. The ^1^H NMR spectra reveal, in particular, the presence of two quartettes representing the AB part of an ABX system due to the CH_2_ protons of the oxazoline rings which appear in the range 3.30–3.71 ppm, and multiplets at 5.16–6.00 ppm, each of which contains one proton, assignable to the isoxazoline CH group (part X), as well as signals at 4.83–5.22 ppm attributable to the aliphatic proton CH_2_N. In the ^13 ^C NMR spectra, especially the Dept 135 NMR, the signals of the isoxazoline CH-carbon which appear at about 80 ppm are noted; this confirms the region specificity of the reaction. This last result is in agreement with the literature^47^^,^^48^.

### 3.3. Synthesis of new molecules containing 1,2,3-triazole linked to 6-nitroindazole derivatives

**Scheme 3.**
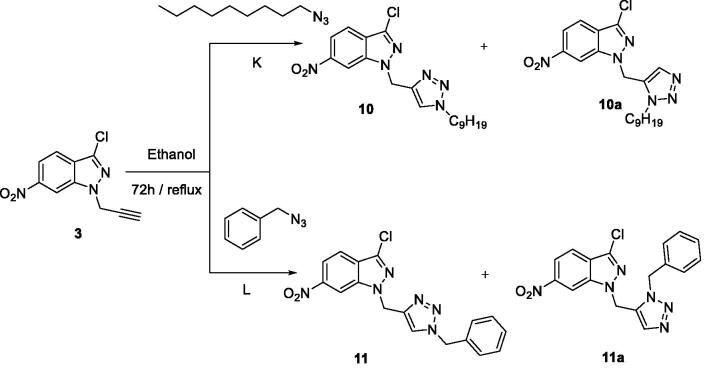
Synthesis of new 1,2,3-triazolyl methyl-6-nitro-1*H*-indazole derivatives.

Given the importance that 1,2,3-triazoles bring to the biological and therapeutic domains^47^, and to broaden this class of compounds, it seemed interesting to combine the 1,2,3-triazole motif with 6-nitroindazoles via a 1,3-dipolar cyclo-addition reaction. The action of azides **K** and **L** on **3** was first examined, according to the Huisgen method (refluxing ethanol for 3 days). Two regioisomers were isolated (Scheme 3) with a yield of 19% for the regioisomer triazole-1,5 (**10** and **10a**) and a yield of 57% for the regioisomer triazole-1,4 (**11** and **11a**). The two 1,4 and 1,5 disubstituted 1,2,3-triazole isomers have been separated by chromatography on silica gel column [eluent: ethyl acetate/hexane (1/9)].

**Scheme 4.**
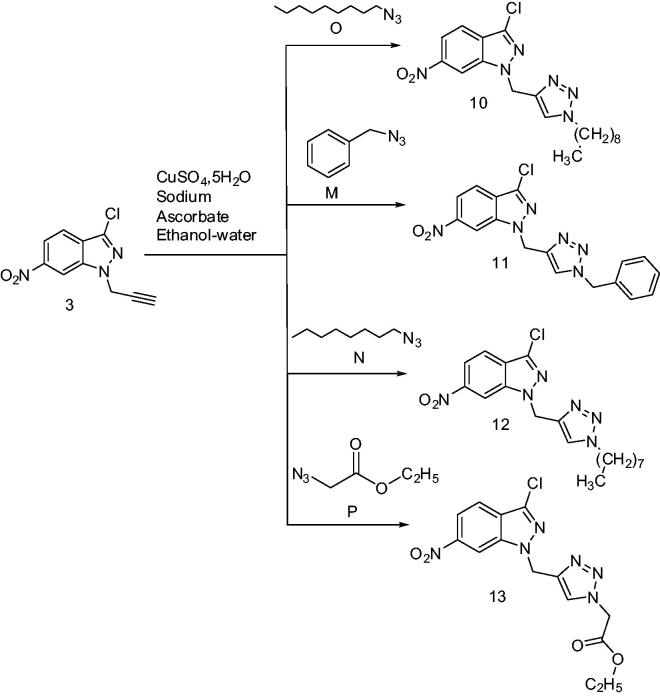
Synthesis of new 1,2,3-triazolylmethyl-6-nitro-1*H*-indazole derivatives under catalytic condition.

The structures of the two products **10a** and **11a** were identified based on the ^1^H NMR spectral data where the H5 protons resonate at about 8.4 ppm. The reverse sense of the cycloaddition would give triazolic H4 protons which would resonate at about 7.5 ppm as previously described in the literature and as we have even found a compound whose proton H4 resonates at 7.25 ppm. Despite the successful separation noted above, the pair of regioisomers, i.e. triazole-1,4 and triazole-1,5 was often difficult to separate by chromatography on silica gel. In a search for a more regiospecific route to the desired compounds, click chemistry [Copper-Catalyzed Azide-Alkyne Cycloaddition (CuAAC)] was utilised, as described in the literature^49–52^. Reacting the dipolarophile **3**, with azides **O**, **M**, **N**, and **P**, in the presence of a Cu(I) catalyst allowed the selective synthesis of the regioisomer triazole-1,4 with yields ranging from 82 to 90%(Scheme 4).

All these products were fully characterised by ^1^H and ^13 ^C NMR (see experimental and calculated methods). The ^1^H NMR spectra in DMSO-d6 of **10–13** show singlet resonances at 4.33 (**10**), 4.49 (**11**), 4.55 (**12**), 4.34 (**12**), and 4.37 (**13**) ppm which are assigned to the two protons of the methylene group linked to the nitrogen atom of the 6-nitroindazole moiety and additional singlets at 7.93 (**10**), 8.01 (**11**), 7.99 (**12**), and 8.39 (**13**) ppm corresponding to the proton in position 5 of the 1,2,3-triazole ring. The ^13 ^C NMR spectra of **10–13** show signals of the two methylene groups linked to the nitrogen atom in position 3 of the bicyclic system at 40.78 (**10**), 41.57 (**11**), 41.42 (**12**), and 40.99 (**13**) ppm. These results are in good agreement with those reported in the literature^53–55^.

### 3.4. Synthesis of isoxazoles bound to 6-nitroindazole derivatives

**Scheme 5.**
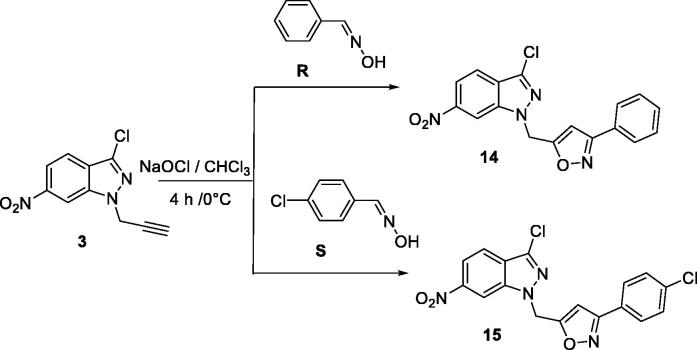
Synthesis of new isoxazole-6-nitro-1*H*-indazole derivatives.

The reaction of the propargyl compound **3** with the aryloxides generated *in situ* from the aldoximes (**R**, and **S**) and the corresponding isoxazoles (**14** and **15**) in each case has led to a single oxazole-structured regioisomer (Scheme 5).

The ^1^H NMR spectra of **14** and **15** have a signal at 6.8 ppm attributable to the isoxazole proton at position 4^55^. The ^13 ^C NMR spectra show, in particular, signals at 105 ppm due to isoxazolic carbons at position 4.

### 3.5. Antileishmanial activity

**Figure 1.**
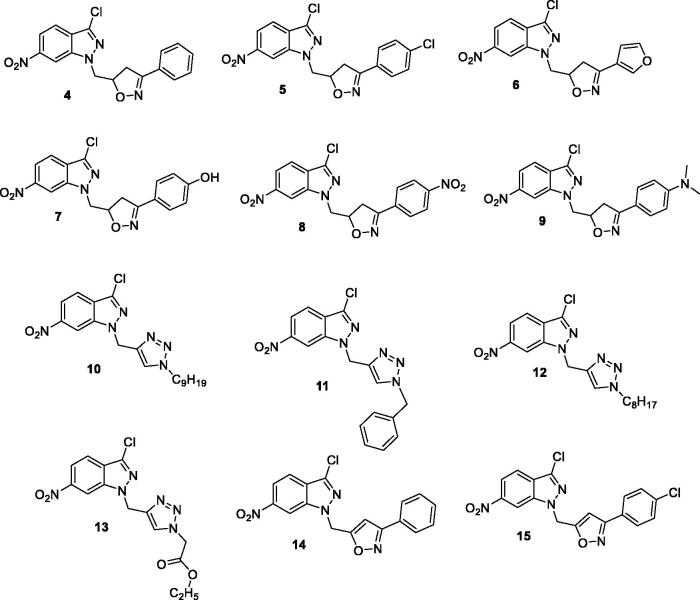
The molecules tested against leishmanias.

The present study was carried out to determine the *in vitro* antileishmanial activity of the 3-chloro-6-nitro-1*H*-indazole derivatives (4–15) against three *Leishmania* species (*L. infantum* (MHOM/MA/1998/LVTA), *L. tropica* (MHOM/MA/2010/LCTIOK-4), and *L. major* (MHOM/MA/2009/LCER19-09), following the established experimental and theoretical procedures. Promastigote strains were exposed to increasing concentrations of compounds (4–15**)** (Figure 1) (analyzed by the MTT assay as described below) and different inhibitory activities towards the three promastigote species were observed. The inhibitory concentration (IC_50_) was measured in µg/ml and compared with that of Glucantime as a reference standard. Glucantime is considered as first-line therapy for cutaneous leishmaniasis. The above-mentioned three *Leishmania* species are the causative agent of *Leishmania* species that why Glucantime was used as a control. The moderate activity of Glucantime compared to the synthesised drugs maybe because of resistance emergence against the control drug.

The antileishmanial activities of all the compounds are summarised in Table 1.

**Table 1.** Inhibitory concentration (IC_50_) values in μM of twelve synthetic compounds (**4–15**) against promastigotes.

Molecules	*L. major*	*L. tropica*	*L. infantum*
4	>500	>500	15.53
5	>500	>500	11.23
6	>500	>500	>500
7	>500	>500	328.59
8	>500	>500	280.84
9	>500	>500	>500
10	>500	>500	174.12
11	>500	213.44	16.85
12	>500	>500	102.03
13	106.72	>500	308.93
14	>500	>500	>500
15	>500	>500	>500
Control Glucantime^®^	>500	>500	>500

Lower IC50 indicates concentration of the compounds required to kill the organism by 50%.

**Figure 2.**
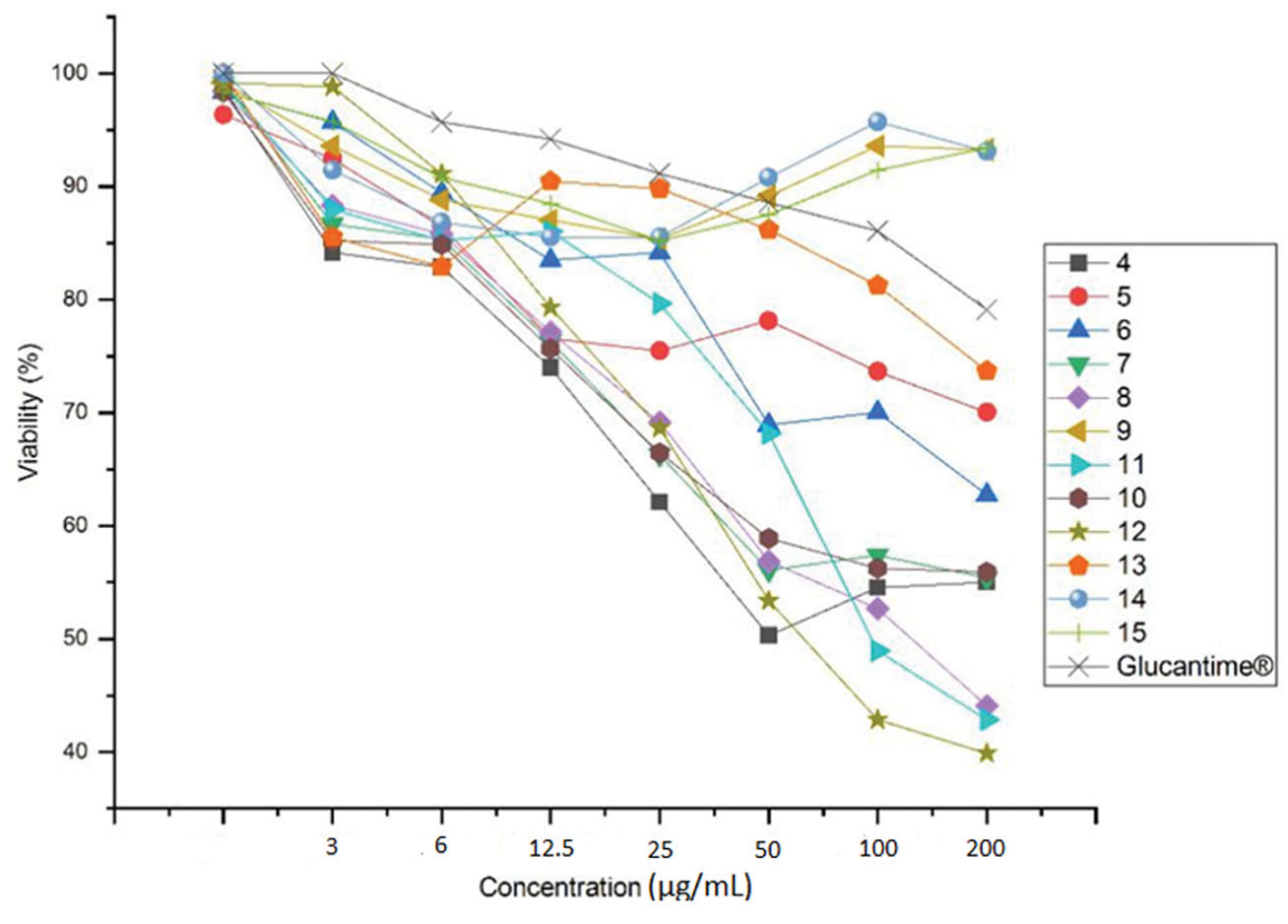
Antileishmanial activity of **4–15** against the main promastigotes of *L. major*.

**Figure 3.**
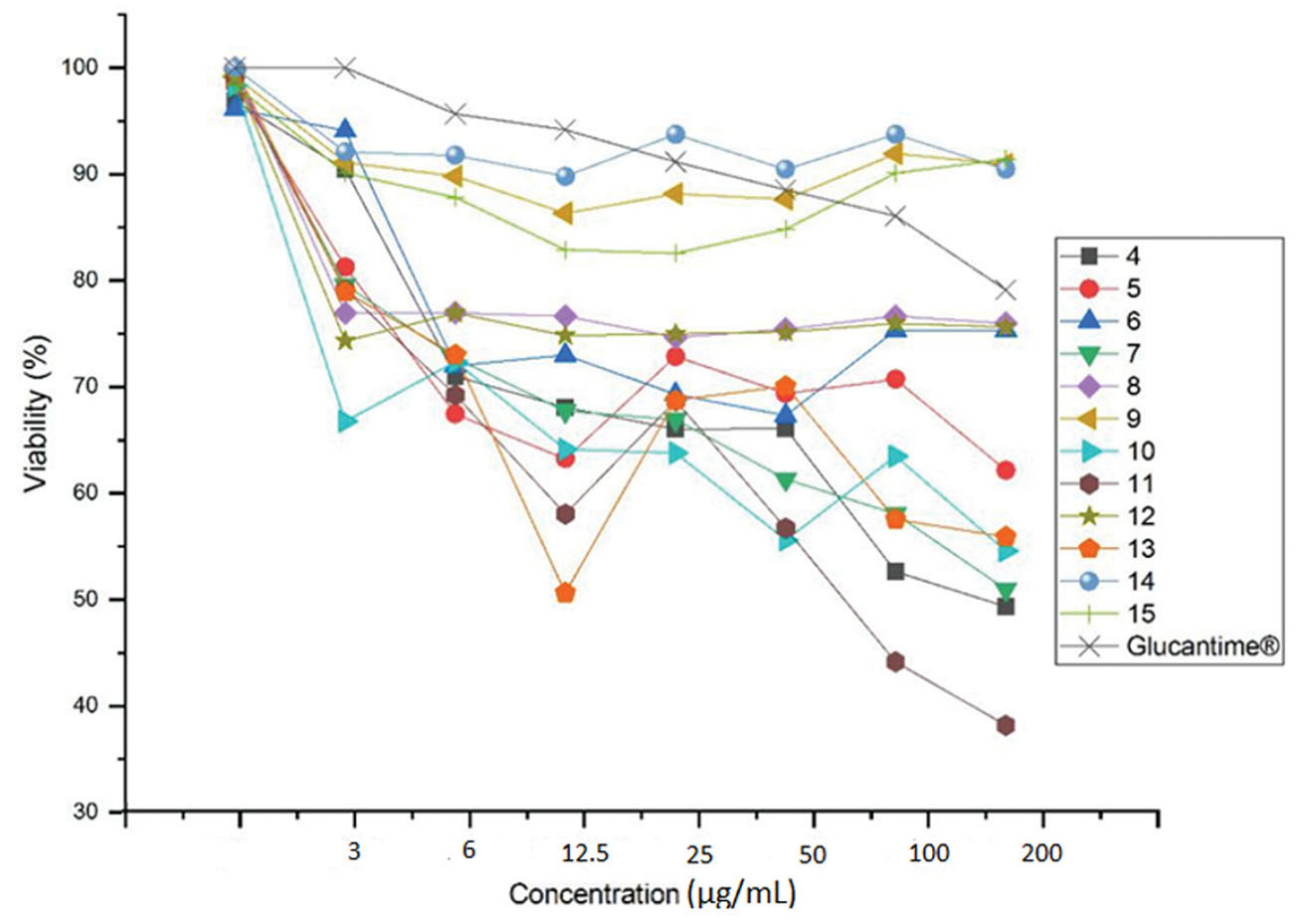
The anti-leishmanial activity of **4–15** against *L. tropica*.

**Figure 4.**
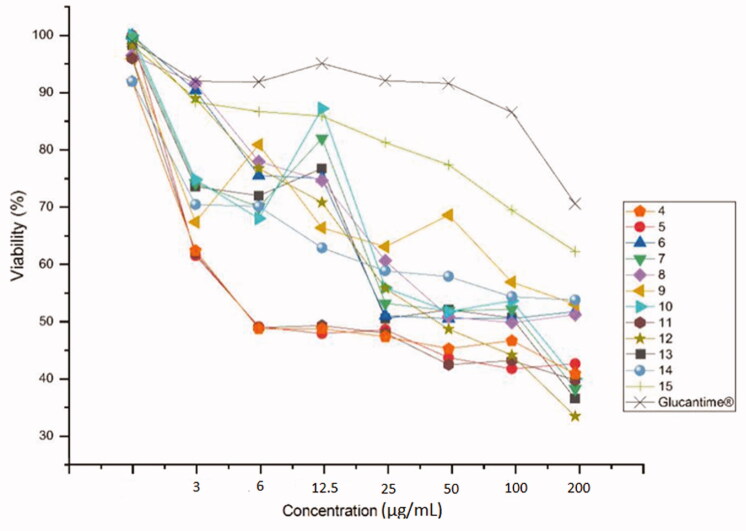
The anti-leishmanial activity of **4–15** against *L. infantum*.

The most effective against *L. major* for a compound embodying a triazole obtained by cycloaddition with azide **P** is **13** at concentration IC_50_ = 38 µM (Table 1). Against *L. tropica*, only **11** and **13** containing a triazole obtained by cycloaddition with azide **M** and **P** had significant efficacy at IC_50_ = 76 and 186 µM (Table 1). All the products particularly those obtained by cycloaddition with dipoles **C**, **D**, **F**, **G**, **O**, **M**, **N**, and **P**, i.e. **4**, **5**, **7**, **8**, **10**, **11**, **12**, and **13**, possess a strong inhibitory activity against *L. infantum*: IC_50_ = 5.53, 4, 117, 100, 62, 6, 36.33, and 110 µM, respectively (Table 1). Finally, **14** and **15**, obtained by cycloaddition with azide **R** and **S**, displayed no growth inhibition towards the three species of Leishmania. Further, increasing concentration of the compounds *vs.* of viability percentage of the organisms is presented in Figures 2–4. Briefly, compound **12** is revealed to show a promising inhibition effect on the viability of *L. major* and *L. infantum* whereas *L. tropica* is most lethal to *L. tropica* at a concentration of µg/ml.

Generally, some molecular characteristics of the 1,2,3-triazoles or oxazolidines can also be used as linkers and show bio-esoteric effects on peptide linkages, aromatic rings, and double bonds. Some unique features like hydrogen bond formation, dipole-dipole, and π stacking interactions of triazole compounds have increased their importance in the field of medicinal chemistry as they bind with the biological targets with high affinity due to their improved solubility.

**Figure 5.**
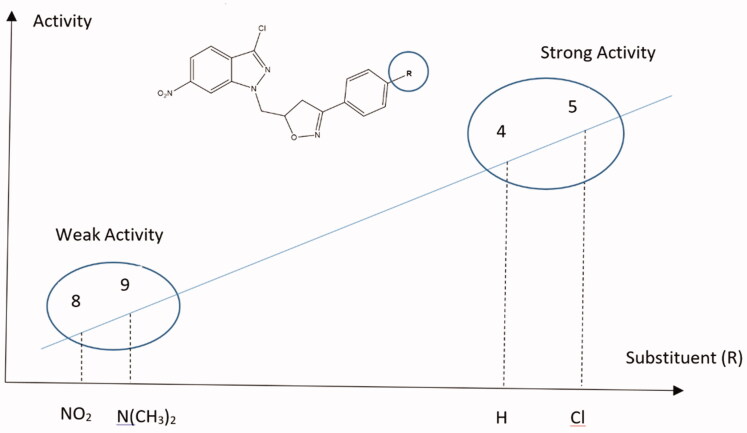
The role of the substituent functional groups on SAR of the synthesised compounds.

**Figure 6.**
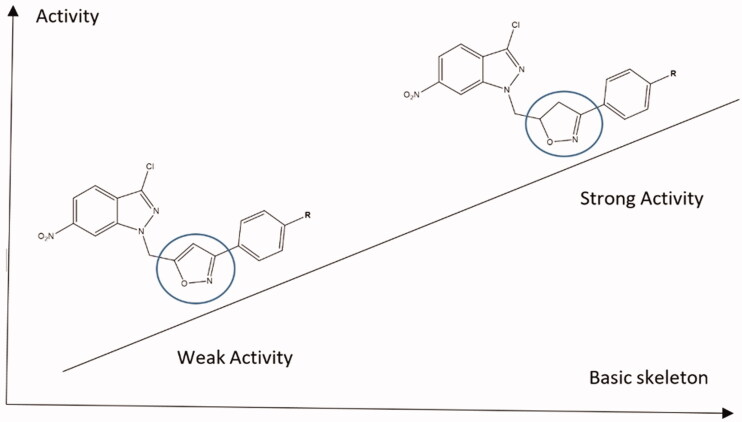
The role of the basic skeleton on SAR of the synthesised compounds.

Some of the important features of the structure-activity relationship of the synthesised compounds are displayed in Figures 5, 6. Figure 5 represents compounds **4**, **5**, **8**, and **9** that have the same basic skeleton and differ only with the nature of the substituent group (R), while Figure 6 shows the subdivision of the compounds to their basic skeletons, two subclasses were considered, the first group consists of compounds **4**, **5**, **8**, and **9**; the second group consists of compounds **14** and **15**. It is evident from Figure 6, the role of 4,5-dihydroisoxazole in increasing the activity of the synthesised compounds. However, Figure 5 indicates that this increase is also dependent on the nature of the substituent group (Figure 5).

### 3.6. Molecular docking results

3-chloro-6-nitro-1*H*-indazole derivatives were tested *in vitro* for antileishmanial activity against three *Leishmania* species *L. infantum*, *L. tropica*, and *L. major* (Table 1). Antileishmanial tests illustrated that the inhibitory potency of 3-chloro-6-nitro-1*H*-indazole derivatives depends on the *Leishmania* species. For instance, *L. tropica* and *L. major* show no activity towards the compounds (except **13** for L. major; and **11** and **13** for L. tropica). However, for *L. Infantum*, seven derivatives (**4**, **5**, **7**, and **10**–**13**) exhibit strong to moderate activity. To understand the observed activities and the higher activity of **4**, **5**, **11**, and **13**, molecular docking studies have been carried out to shed light on the binding modes between the docked compounds (**4**, **5**, **11**, and **13**) and the active residues of the targeted TryR from *L. infantum*. The binding energies of the stable complexes ligand-TryR, the number of established intermolecular hydrogen bonds between the docked ligands (**4**, **5**, **11**, and **13**), and the active site residues of trypanothione reductase are determined (Table 2).

**Table 2.** Docking binding energies, number of hydrogen bonds and number of closest residues to the docked ligands (**4**, **5**, **11**, and **13**) within the active binding site of the targeted TryR.

Name of synthesised derivatives	Free binding energy (kcal/mol)	H-bonds (HBs)	Number of closest residues to the docked ligand into the active site	IC50 (µM)
**4**	−9.33	0	8	15.33
**5**	−10.10	0	9	11.23
**11**	−9.26	1	8	16.85
**13**	−9.00	5	9	308.93

**Figure 7.**
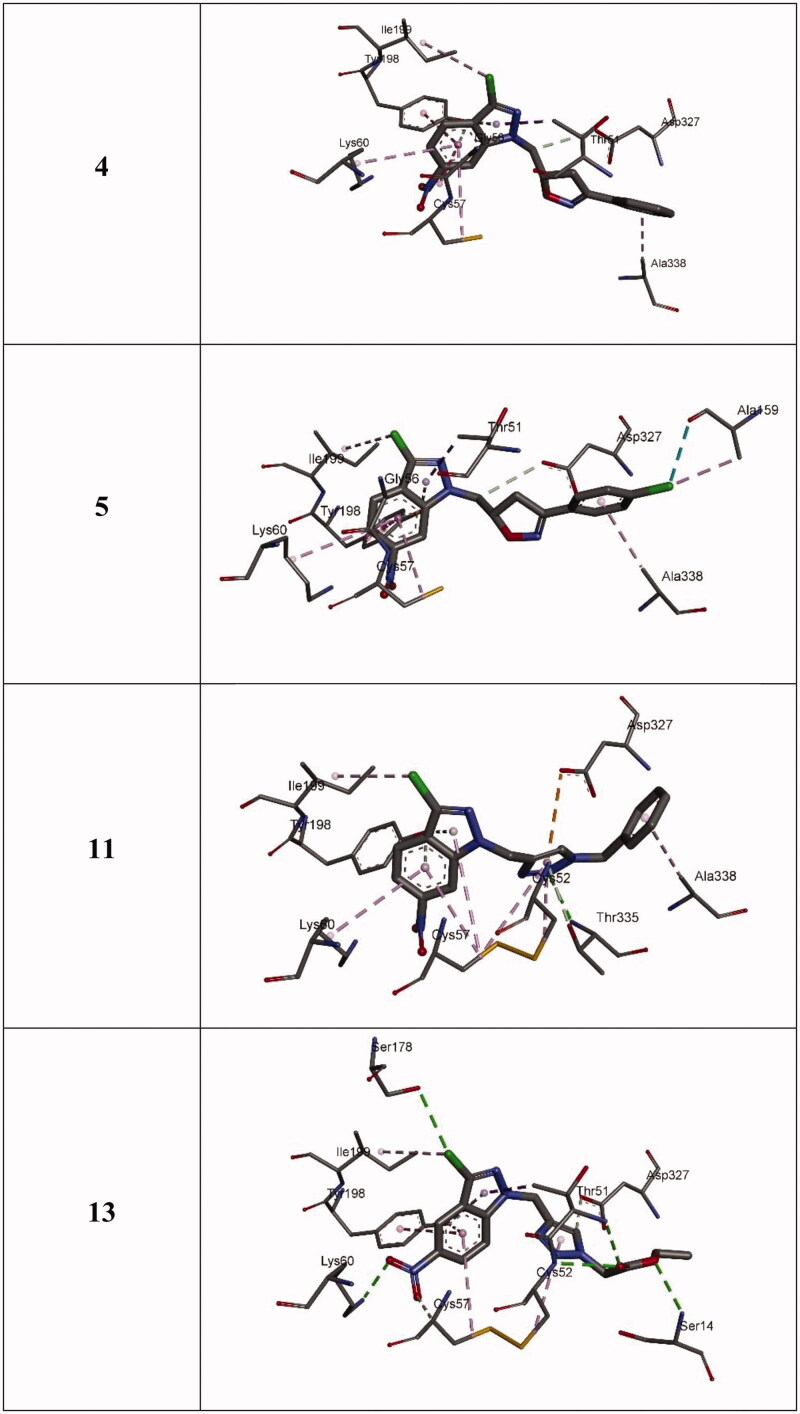
3 D closest interactions between active residues of TryR and the docked synthesised derivatives **4**, **5**, **11**, and **13**.

**Figure 8.**
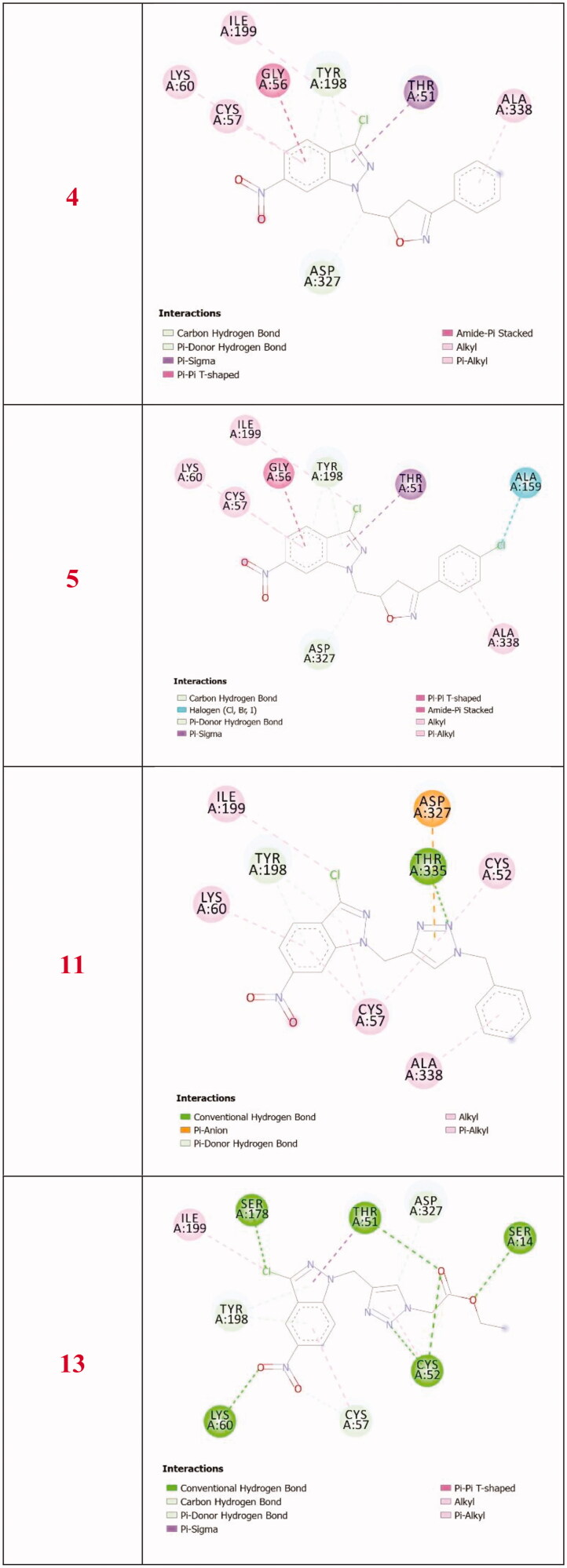
2 D closest interactions between active residues of TryR and the docked molecules **4**, **5**, **11**, and **13**.

All complexes formed between the docked compounds (**4**, **5**, **11**, and **13**) and the active site of TryR showed negative binding energies which indicates that the docking of **4**, **5**, **11**, and **13** is thermodynamically favourable. The inhibition studies showed that **5** is more active than **4** and this is in accordance with the docking results which showed that the docked 5-TryR complex is more stable than the 4-TryR complex (Table 2). **4** and **5** differ only by the chlorine at the *para*-position of the phenyl group of the isoxazole (Figure 1). Both complexes 5-TryR and 4-TryR show similar interactions with the active amino acids of TryR (Figures 7, 8). The higher activity of **5** compared to **4** is mainly due to the presence of the chlorine at the *para*-position of the phenyl group of the isoxazole which interacts with the CO group of ALA159 at a distance of 3.16 Å thereby providing an additional stabilisation to the complex. This may also explain the higher activity of **5** contrasted with **11** and **13** since the latter lacks the 4-chlorophenyl substituent. Indeed, the highest binding energy is obtained with **5**.

### 3.7. Molecular dynamics studies

**Figure 9.**
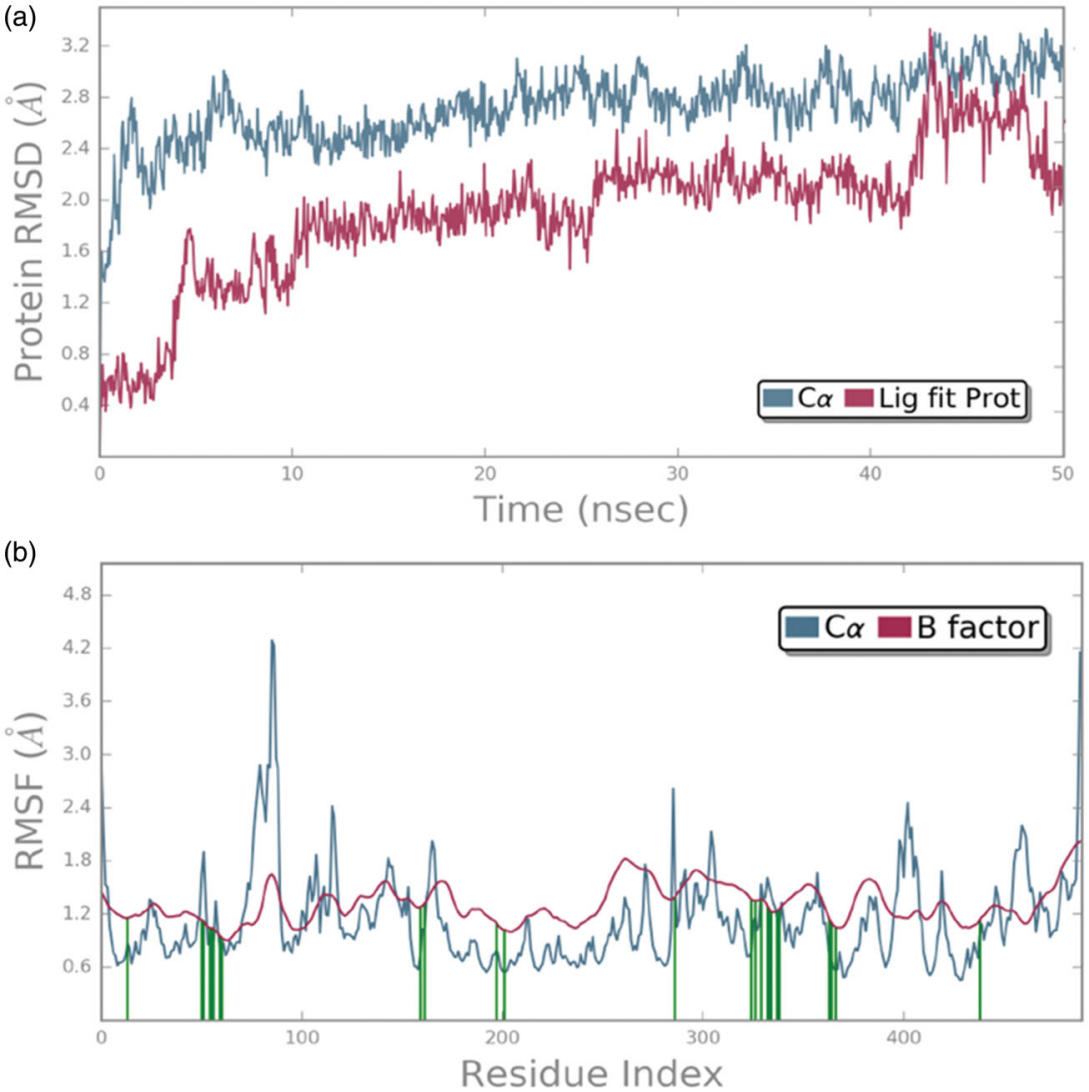
The (a) RMSD (b) RMSF analysis plot of TryR-**13** complex system.

**Figure 10.**
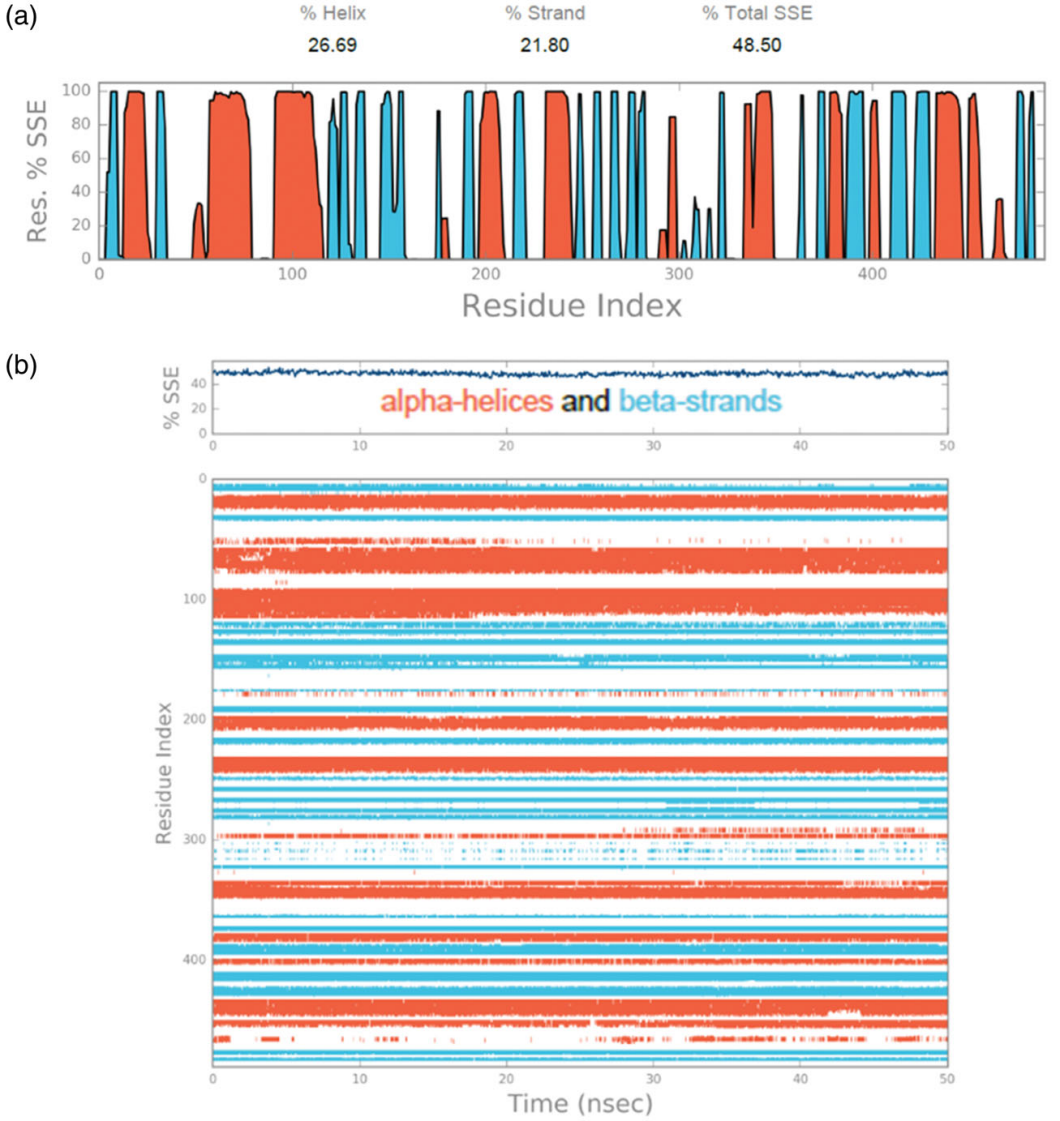
(a) SSE distribution by residue index throughout the TryR structure (b) SSE composition for each trajectory frame over the course of the simulation TryR secondary structure elements (SSE) like alpha helices and beta strands are monitored throughout the simulation.

**Figure 11.**
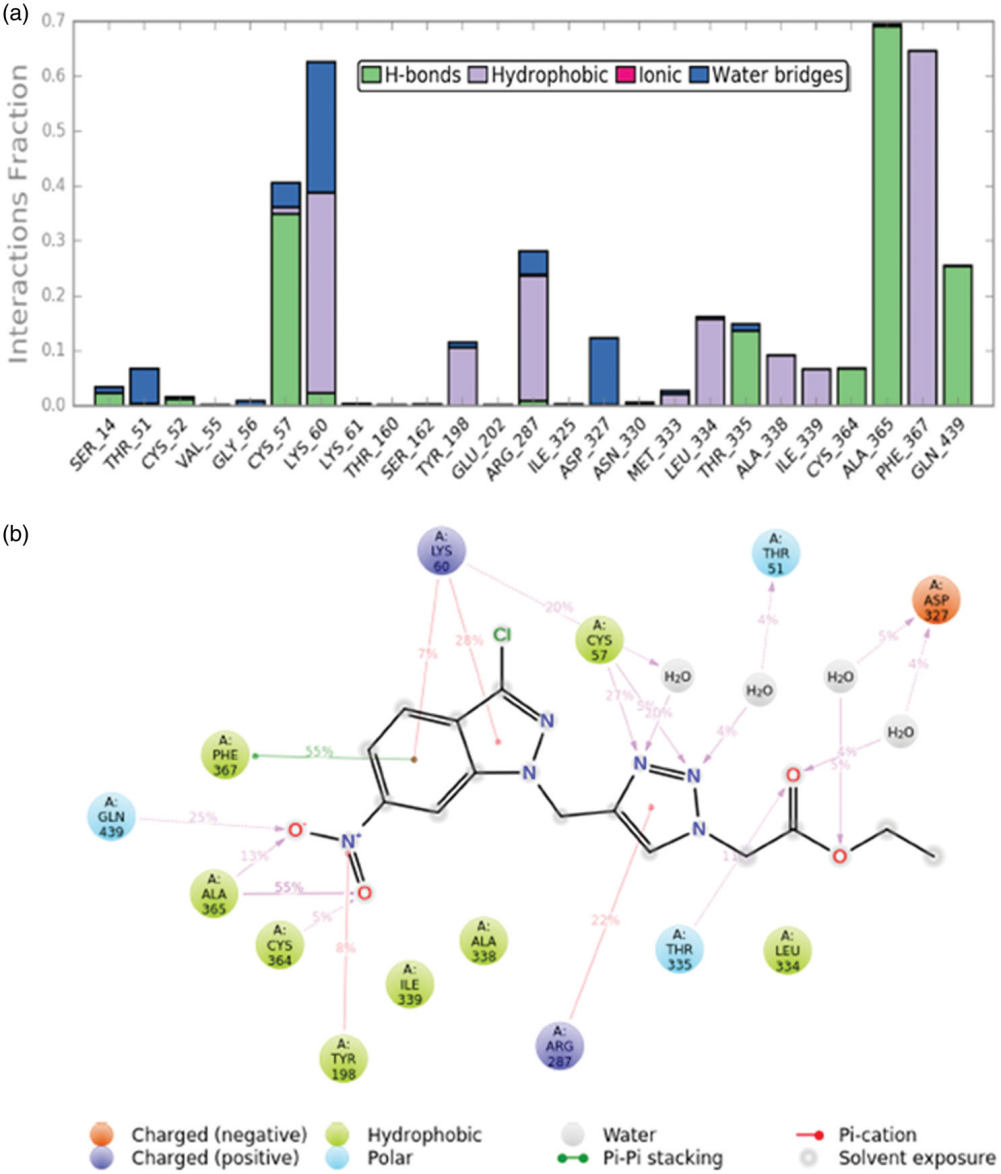
(a) TryR-**13** contacts during 50 ns simulation (b) a schematic representation of the interaction between **13** and the TryR residues.

The molecular dynamics simulations were performed to understand the stability of the small molecules in the protein complex^56–61^. After the docking studies, the best binding pose was subjected to molecular dynamics simulations to analyse the stability of **13** in the TryR complex system. The RMSD and RMSF were measured initially to explore the protein-ligand conformation and structural fluctuation during the 50 ns simulation. All protein frames are first adjusted on the reference frame backbone and after that, the RMSD is determined dependent on the molecule selected. Monitoring the RMSD of the protein can give insight into its basic adaptation all through the simulation. The RMSD (Figure 9(a)) result suggests that the TryR-13 complex system is perfectly acceptable for small, globular proteins due to the fact that our simulation can converge (the RMSD values stabilise around a fixed value in the order of 1–3 Å). Ligand RMSD (Lig fit Prot) indicates how stable **13** is with respect to the TryR binding pocket. In the above plot, “Lig fit Prot” shows the RMSD (Figure 9(a)) when the TryR-13 complex is first aligned on the TryR reference backbone and then the RMSD of the heavy atoms on **13** is measured. The values observed are significantly lower than the RMSD of TryR which indicates that **13** could form a stable complex with TryR. The RMSF is used to characterise local changes along the protein chain. In Figure 9(b), peaks indicate which areas of TryR fluctuate the most during the simulation. Typically, we observe that the tails (N- and C-terminal) fluctuate more than any other part of the TryR system. Secondary structure elements (SSE) like alpha helices and beta strands are usually more rigid than the unstructured part of TryR, and thus vary less than the loop regions. TryR residues that interact with **13** are marked with green-colored vertical bars. Moreover, Figure 10(a) represents SSE distribution by residue index throughout the protein structure. Figure 10(b) outlines the SSE composition for each trajectory frame over the course of the simulation, and the plot at the bottom monitors each residue and its SSE assignment over time. The communications between TryR and **13** can be checked all during the simulation. This binding interaction can be sorted by type and summarised, as appeared in Figure 11. Protein-ligand connections (or “contacts”) are classified into four kinds: (1) hydrogen bonds, (2) hydrophobic, (3) ionic, and (4) water bridges. Every interaction type contains progressively explicit subtypes, which can be investigated by the simulation interactions diagram. The stacked bar diagrams are standardised through the span of the direction: for instance, an estimation of 0.7 proposes that the particular interaction is kept up in 70% of the recreation time. Qualities over 1.0 are conceivable as some protein buildup may make different contacts with the ligand by the same subtype. The overall results suggest that **13** was stable in the TryR system during the simulation.

### 3.8. MM/GBSA binding free energy estimation

**Figure 12.**
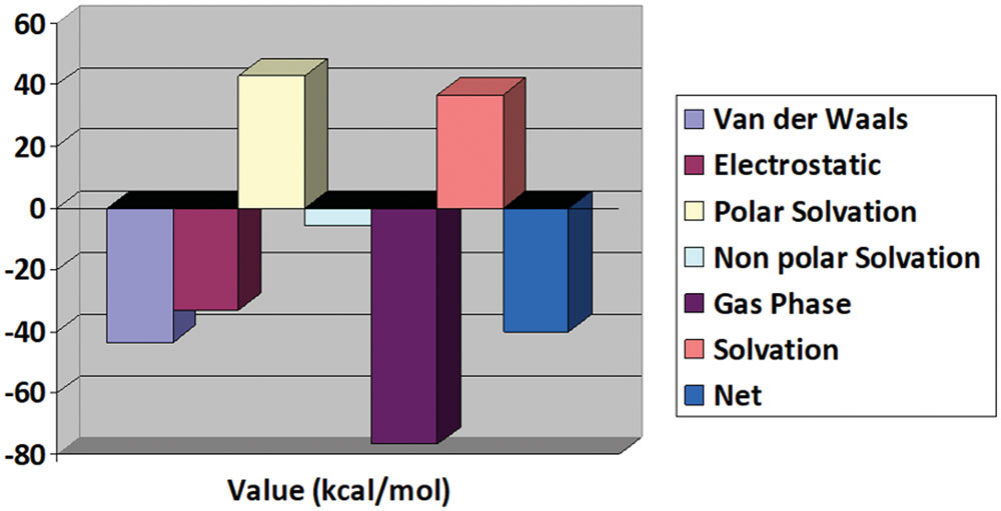
Different MM/GBSA binding free energies of compound 13-TryR complex.

The binding affinity of compound **13** for the TryR enzyme was re-validated by a more sophisticated approach of MM/GBSA binding free energy than the traditional docking score function. The compound was revealed to show good docked stability with the enzyme by scoring a very low net binding energy score of −40.02 kcal/mol. The intermolecular complex interactions are dominated by both Van der Waals and electrostatic energy as well as non-polar solvation energy. The polar solvation energy though found non-contributing to the system. Overall, like previous analysis, MM/GBSA also support the biological potency of compound 13 against the leishmanial parasite. The different binding energies of the systems are presented in Figure 12.

### 3.9. DFT-based structure-activity analysis

According to the aforementioned comparison of the anti-leishmanial activities of the title compounds, we can expect that a compound’s anti-leishmanial activity has some relationship with its molecular properties. Indeed, recently, Nascimento et al. performed a DFT-B3LYP study on nineteen neolignan derivatives which could be categorised into two groups active and inactive according to their anti-leishmanial efficiencies. An assessment of the molecular electrostatic potentials (MEPs) of the nineteen compounds showed the active compounds have much more intense regions of negative electrostatic potentials than the inactive ones^62^. Similarly, Pinheiro and co-workers synthesised eleven 5-(4,5-dihydro-1*H*-imidazol-2-yl)-4-(arylamino)thieno[2,3-*b*]pyridine derivatives as new anti-leishmanial compounds. Their theoretical analyses also suggested that the anti-leishmanial activities detected in the eleven compounds correlate with their MEPs^63^. Therefore, in this study, we also investigate the MEPs of **4–15** (Figure 12) to rationalise their differences in their anti-leishmanial activity. Exploiting the full-density matrix, the total densities of **4–15** were generated and the respective MEP was mapped on their surfaces (Figure 13)^58^^,^^59^. MEPs are fundamental measures of the strength of the nearby charges, nuclei, and electrons at a particular position and thus enable us to visualise the charge distributions and charge-related properties of molecules. To make the electrostatic potential data easy to interpret, visual representation with a colour spectrum is used with red representing the lowest electrostatic potential value and blue the highest. Beneath the molecular designation is its B3LYP-optimised geometry^57–59^. Not as expected, **13** did not have the most negative electrostatic potentials among the group. However, we see from Figure 10 that **10**, **11**, **12**, and **13** are more bent in geometry than the other compounds. Hence, we propose that there may be some interactions between the core, 3-chloro-6-nitro-1*H*-indazole, and the side chain of the triazolic derivatives in **10**, **11**, **12**, and **13**. These interactions should influence their anti-leishmanial activities. So we are doing further research in two directions: (1) the correctness of the theoretical level, and (2) the interactions between the core, 3-chloro-6-nitro-1*H*-indazole, and the side chain of the triazolic derivatives in **10**, **11**, **12**, and **13**^64^.

Figure 13.MEPs of the tested compounds in this study (isovalue = 0.0004; the atomic colour: carbon in grey, chlorine in green, hydrogen in white, nitrogen in blue, and oxygen in red).

**Figure:**
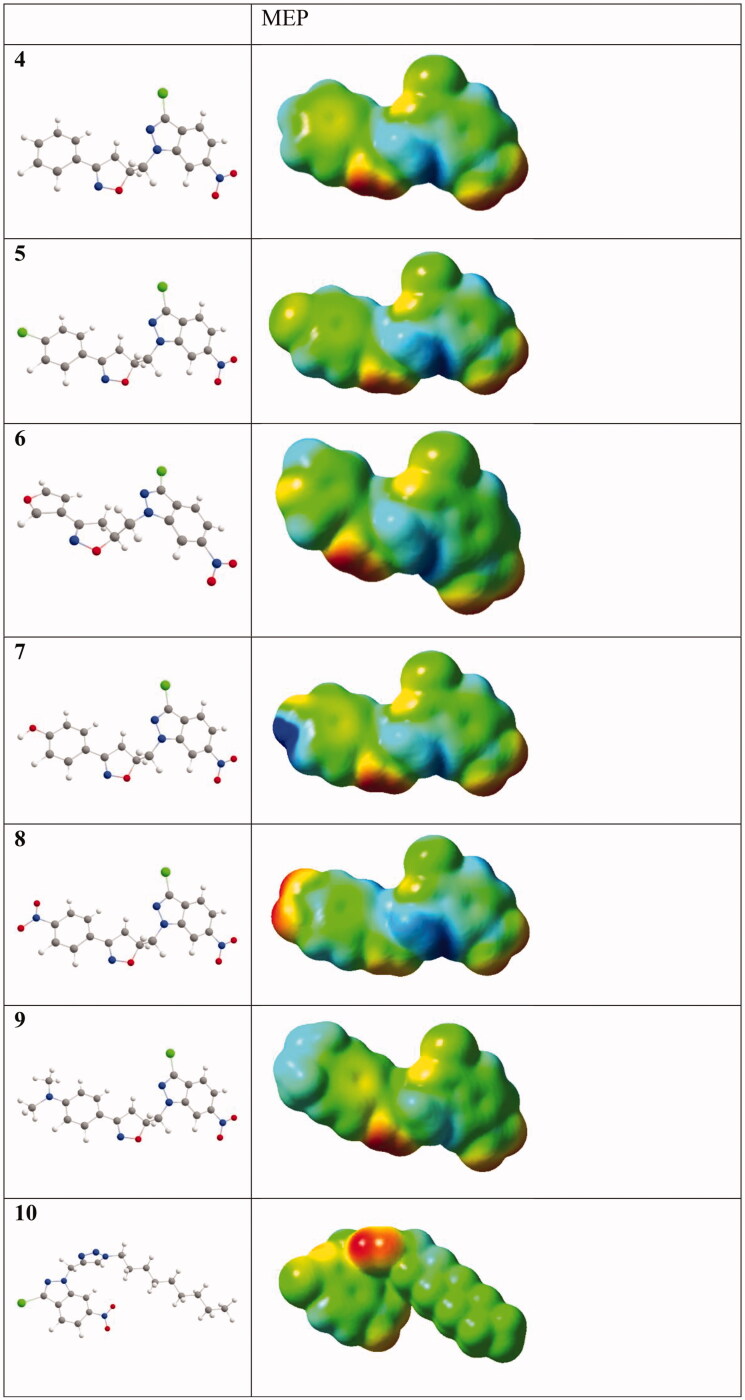

**Figure:**
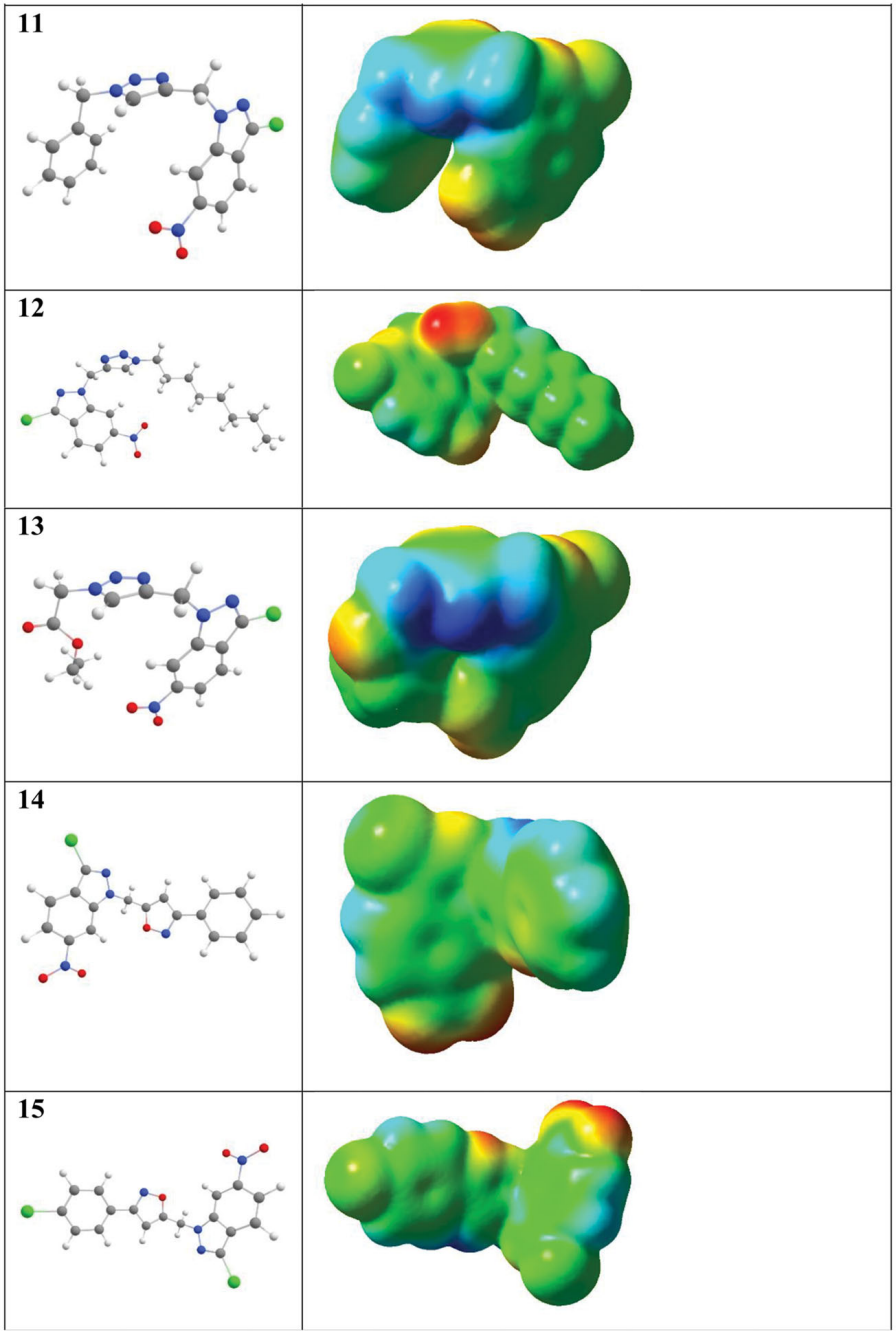

## 4. Conclusions

In the present work, synthesis of new heterocyclic systems originated from 3-chloro-6-nitro-1*H*-indazole was carried out in satisfactory yields by cycloaddition reactions under thermal and catalytic conditions (CuI). The results showed a periselectivity and regioselectivity as a function of the dipole (azides **F**, **G**, and **H**) employed. Additionally, it was shown that indazole derivatives have biological potency against three species of Leishmania and can be utilised as an excellent inhibitor for the parasite of leishmania. Moreover, in comparison of the findings of all the derivatives with the Glucantime as a reference standard, it was observed that indazole could be exploited as a useful source in the discovery of new antileishmania drugs. The inhibitory efficacy of indazole derivatives was changed significantly when the rings associated with indazole were changed. Thus compounds containing triazole, proved more efficient in inhibition than compounds containing oxazoline while those containing oxazole had the lowest effectiveness. This may be due to the interactions between the core, 3-chloro-6-nitro-1*H*-indazole, and the side chain of the triazolic derivatives. The molecular docking, molecular dynamics, and MM/GBSA binding free energy results were in good agreement with the experimental studies. Triazoles are reported as a potent inhibitor of cytochrome P450 particularly CYP3A4, which often leads to toxic effects and drug interactions when used in combination with drugs that are dependent for metabolism on cytochrome P450 enzymes. Therefore care should be taken while using it in combination with other drugs to avoid this phenomenon.

## Supplementary Material

Supplemental Material
