# Supplementary material for: Novel 3-chloro-6-nitro-1H-indazole derivatives as promising antileishmanial candidates: synthesis, biological activity, and molecular modelling studies
Source: J Enzyme Inhib Med Chem. 2021 Dec 11;37(1):151–67. doi: 10.1080/14756366.2021.1995380 (PMC8667887; doi:10.1080/14756366.2021.1995380)
Supplement: Supplemental Material [file IENZ_A_1995380_SM9920.pdf]

Novel 3-chloro-6-nitro-1*H*-indazole Derivatives as promising Antileishmanial candidates: synthesis, biological activity and molecular modeling studies

Mohamed Mokhtar Mohamed Abdelahi,[1,#] Youness El Bakri,[1,2*#] Chin-Hung Lai,[3,4] Subramani Karthikeyan,[5] El Hassane Anouar[6], Sajjad Ahmad[7], Mohammed Benchidmi[1], Joel T. Mague[8], Jelena Popović-Djordjević[9], Souraya Goumri-Said[10,*]

[1]*Laboratoire de Chimie Organique Hétérocyclique, Centre de Recherche des Sciences des Médicaments, Pôle de Compétences Pharmacochimie, URAC 21, Faculté des Sciences, Mohammed V University Rabat, Avenue Ibn Battouta, BP 1014, Rabat, Morocco*
[2.] *Department of Theoretical and Applied Chemistry, South Ural State University, Lenin prospect 76, Chelyabinsk, 454080, Russian Federation.*

[3.] *Department of Medical Applied Chemistry, Chung Shan Medical University, Taichung 40241, Taiwan*
[4]*Department of Medical Education, Chung Shan Medical University Hospital, Taichung 40241, Taiwan*
[5] *G.S Gill Research Institute, Guru Nanak College, Chennai 600042, India*
[6]*Department of Chemistry, College of Sciences and Humanities in Al-Kharj, Prince Sattam bin Abdulaziz University, Al-Kharj 11942, Saudi Arabia*

[7.] *Department of Health and Biological Sciences, Abasyn University, Peshawar 25000, Pakistan*
[8] *Department of Chemistry, Tulane University, New Orleans, LA 70118, USA*
[9]University of Belgrade, Faculty of Agriculture, Department for Chemistry and Biochemistry, Nemanjina 6, 11080 Belgrade, Serbia
[10]College of Science, Physics Department, Alfaisal University, P.O. Box 50927, Riyadh, 11533, Saudi Arabia

Supporting information

1. General procedure for synthesis of compounds 4, 5, 6, 7, 8, and 9

To a solution of 0.01 mol of 1-allyl-3-chloro-6-nitro-1H-indazole 2 and 0.02 mol of oxime in 40 mL of dichloromethane with magnetic stirring at 0 ˚C, 20 ml of a bleach solution was added dropwise. The reaction was followed by thin-layer chromatography until the starting material had disappeared. The organic phases were decanted, washed twice with 20 mL of water and dried over magnesium sulfate. After filtration and removal of the solvent under reduced pressure, the products were purified by chromatography on a silica column, eluting with a mixture of hexane and ethyl acetate (90/10). We reported in the supplementary information, all the details of synthesis and amount of chemicals used.

5-((3-chloro-6-nitro-1H-indazol-1-yl)methyl)-3-phenyl-4,5-dihydroisoxazole (4)

Brown solid, Yield = 74%. m.p: 175-177 ˚C. ^{1}H NMR (300 MHz, DMSOd_6) : 3.37 (q, J_1 = 9 Hz ; J = 18 Hz 2 H CH), 3.60 (q, J = 9 Hz ; J = 18 HZ 2 H CH), 4.82 (d, J = 6 HZ, 2 H CH_2-N), 5.15-5.20 (m, 1 H CH), 7.40-7.45 (m, 3H CH Ar), 7.57-7.60 (m, 2 H CH Ar), 7.89 (d, J = 9 Hz, 1 H CH Ar), 8.02 (d, J = 9 Hz, 1 H CH Ar), 8.87 (d , J = 3 Hz, 1H CH Ar). ^{13}C NMR (75 MHz, DMSOd_6): 38.05 (CH_2), 52.68 (CH_2), 79.54 (CH), 108.98, 116.63, 120.92, 127.09, 129.27 (CH Ar), 123.43, 130.69, 131.20, 140.84, 147.59, 147.81, 157.33 (C_q). HRMS (ESI) Calculated for $C_{17}H_{13}N_4O_3Cl$: [M+H$^+$] = 356.0709, Found: [M+H$^+$] = 356.0725. Elemental analysis Calculated: C, 57.23%; H, 3.67%; N, 15.70%; O, 13.45%; Cl, 9.94 % Found: C, 57.31%; H, 3.43%; N, 15.78%; O, 13.60%; Cl, 9.88%.

5-((3-chloro-6-nitro-1H-indazol-1-yl)methyl)-3-(4-chlorophenyl)-4,5-dihydroisoxazole (5)

Brown solid, Yield = 62%. m.p: 195-197 ˚C. ^{1}H NMR (300 MHz, DMSOd_6): 3.36 (q, J = 6 Hz; J = 18 Hz 2 H CH), 3.60 (q, J = 6 HZ; J = 18 Hz 2 H CH_2), 4.82 (d, J = 6 Hz, 2 H CH_2), 5.17-5.21 (m, 1 H CH), 7.47 (d, J = 9 Hz, 2 H CH Ar), 7.59 (d, J = 9 Hz, 2 H CH Ar), 7.87 (d, J = 9 Hz, 1 H CHAr), 7.99 (d, J = 9 Hz, 1 H CH Ar), 8.83 (d, J = 1.53 Hz, 1 H CH Ar). ^{13}C NMR (75 MHz, DMSOd_6): 52.76 (CH_2), 79.87 (CH), 108.83, 116.58, 120.88, 123.57, 128.80, 129.29 (CH Ar), 131.06, 133.02, 135.26, 140.92, 147.77, 156.57 (C_q). HRMS (ESI) Calculated for $C_{15}H_{11}N_4O_3Cl_2$: [M+H$^+$] = 390.0302, Found: [M+H$^+$] = 390.0319. Elemental analysis Calculated: C, 52.19%; H, 3.09%; N, 14.32%; O, 12.27%; Cl, 18.12 % Found: C, 52.25%; H, 3.05%; N, 14.39%; O, 12.16%; Cl, 18.15 %.

5-((3-bromo-6-nitro-1H-indazol-1-yl)methyl)-3-(furan-2-yl)-4,5-dihydroisoxazole (6)

Brown solid, Yield = 62%. m.p: 151-153 ˚C. ^{1}H NMR (300 MHz, DMSOd_6): 3.28 (dd, J = 6 Hz; J = 18 HZ 2 H CH), 3.53 (dd, J = 12 Hz; J_2 = 30 Hz 2 H CH_2), 4.82 (d, J = 6 Hz, 2 H CH_2), 5.11-5.16 (m, 1 H CH), 6.60 (d, J = 6 Hz, 1H CH), 6.88 (d, J = 6 Hz, 1H CH), 7.78 (d, J = 15 Hz, 1 H CH Ar), 7.95 (d, J = 9 Hz 1 H CH Ar), 8.84 (d, J = 1.53 Hz, 1 H). ^{13}C NMR (75 MHz, DMSOd_6): 38.1 (CH_2), 52.48 (CH_2), 79.24 (CH), 108.72, 109.71, 112.45, 113.78, 116.6, 121.47, 126.16 (CH Ar), 140.65, 143.20, 144.43, 145.77, 147.54, 149.23 (C_q). HRMS (ESI) Calculated for $C_{15}H_{11}N_4O_4Cl$: [M+H$^+$] = 346.0512, Found: [M+H$^+$] = 346.0531. Elemental analysis Calculated: C, 51.96%; H, 3.20%; N, 16.16%; O, 18.46%; Cl, 10.23 % Found: C, 52.03%; H, 3.26%; N, 16.02%; O, 18.51%; Cl, 10.18 %.

4-(5-((3-chloro-6-nitro-1H-indazol-1-yl)methyl)-4,5-dihydroisoxazol-3-yl)phenol (7)

Brown solid, Yield = 78%. m.p: 180-182 ˚C. ^{1}H NMR (300 MHz, DMSOd$_6$): 3.54-3.65 (q, J = 10 Hz; J_2 = 17 HZ 2 H CH), 3.59 (q, J = 12 Hz; J = 17 Hz 2 H CH), 4.84 (m, 2 H CH_2), 5.16-5.26 (m, 1 H CH), 7.41-7.44 (m, 3 H CH Ar), 7.57-7.60 (m, 2 H CH Ar), 7.89 (dd, J = 0.6 Hz ; J = 9 Hz, 1 H CH Ar), 8.02 (dd, J = 1.8 Hz, J = 9 Hz, 1 H), 8.61 (dd, J = 1.8 Hz; J = 0.6 Hz, 1H CH Ar), 9.49 (s, 1 H OH). ^{13}C NMR (75 MHz, DMSOd$_6$): 38.06 (CH_2), 52.57 (CH_2), 79.45 (CH), 107.30, 115.58, 122.85, 124.29, 127.90, 128.19, 129.09 (CH Ar), 134.52, 137.49, 138.54, 146.58, 156.58 (C_q). HRMS (ESI) Calculated for $C_{17}H_{13}N_4O_4Cl$: [M+H$^+$] = 372.0601, Found: [M+H$^+$] = 372.0621. Elemental analysis Calculated: C, 54.78%; H, 3.52%; N, 15.03%; O, 17.17%; Cl, 9.51 % Found: C, 54.86%; H, 3.47%; N, 14.98%; O, 17.11%; Cl, 9.58 %.

5-((3-chloro-6-nitro-1H-indazol-1-yl)methyl)-3-(4-nitrophenyl)-4,5-dihydroisoxazole (8)

Brown solid, Yield = 82%. m.p: 179-181 ˚C. ^{1}H NMR (300 MHz, DMSOd$_6$): 3.32 (q, J = 6 Hz; J = 18 Hz 2 H CH), 3.65 (q, J = 9 Hz; J = 18 Hz 2 H CH), 4.82 (d, J = 4.2 Hz 2 H CH_2), 5.14-5.20 (m, 1 H CH), 7.54-7.58 (m, 2 H CH Ar), 7.58-7.60 (m, 2 H CH Ar), 7.89 (dd, J = 9 Hz; J = 0.6 Hz 1 H CH Ar), 8.02 (dd, J = 9 Hz; J = 1.8 Hz 1 H), 8.78 (t, J = 1.5 Hz 1 H CH Ar). ^{13}C NMR (75 MHz, DMSOd$_6$): 38.56 (CH_2), 51.15 (CH_2), 80.09 (CH), 108.79, 116.58, 120.99, 123.63, 126.95, 129.54 (CH Ar), 124.89, 134.20, 137.20, 144.30, 148.59, 156.78 (C_q). HRMS (ESI) Calculated for $C_{17}H_{12}N_5O_5Cl$: [M+H$^+$] = 401.0571, Found: [M+H$^+$] = 401.0587. Elemental analysis Calculated: C, 50.82%; H, 3.01%; N, 17.43%; O, 19.91%; Cl, 8.82 % Found: C, 50.97%; H, 3.11%; N, 17.29%; O, 19.70%; Cl, 8.93 %.

4-(5-((3-chloro-6-nitro-1H-indazol-1-yl)methyl)-4,5-dihydroisoxazol-3-yl)-N,N-dimethylaniline (9)

Brown solid, Yield = 82%. m.p: 148-150 ˚C. ^{1}H NMR (300 MHz, DMSOd$_6$): 2.82 (q, J = 3Hz; J = 9 HZ 2 H CH), 2.96 (q, J = 12 Hz; J = 9 Hz 2 H CH), 5.17 (m, 1 H CH_2), 5.95-6.04 (m, 1 H CH), 7.57-7.62 (m, 2 H CH Ar), 7.70-7.75 (m, 2 H CH Ar), 8.21-8.25 (m, 2 H CH Ar), 8.36-8.37 (m, 2 H CH Ar), 8.88 (t, J = 1.5 Hz 1 H CH Ar). ^{13}C NMR (75 MHz, DMSOd$_6$): 37.36 (CH_2), 49.02 (2 CH_3), 61.89 (CH_2), 79.95 (CH), 108.46-116.71, 120.90, 123.67, 126.36, 130.35 (CH Ar), 133.38, 140.89, 147.68, 153.19, 156.19 (C_q). HRMS (ESI) Calculated for $C_{19}H_{18}N_5O_3Cl$: [M+H$^+$] = 399.1164, Found: [M+H$^+$] = 399.1178. Elemental analysis Calculated: C, 57.07%; H, 4.54%; N, 17.52%; O, 12.00%; Cl, 8.87 % Found: C,

57.15%; H, 4.41%; N, 17.60%; O, 11.86%; Cl, 8.98 %.

General procedure for synthesis of compounds 10 and 10a

0.08 mol of 3-chloro-6-nitro-1-(prop-2-yn-1-yl)-1H-indazole **3** and 0.16 mol of the azide were placed in a reactor containing 60 mL of absolute ethanol and the mixture was heated at reflux for 72 h. After evaporation of the solvent under reduced pressure, the products were purified by chromatography on a column of silica eluting with a mixture of hexane and ethyl acetate (90/10).

3-chloro-6-nitro-1-((1-nonyl-1H-1,2,3-triazol-4-yl)methyl)-1H-indazole (10)

Brown solid, Yield = 57 %. m.p: 152-154 °C. ^{1}H NMR (300 MHz, DMSOd$_6$): 0.77 (t, J = 6 Hz 3 H CH$_3$), 1.16-1.24 (m, 12 H CH$_2$), 1.71-1.78 (m, 2H CH$_2$), 4.29 (t, J = 9 Hz 2 H CH$_2$), 5.88 (s, 2H CH$_2$), 7.90 (d, J = 9 Hz 1 H CH Ar), 8.02 (d, J = 9 Hz 1 H CH Ar), 8.15 (s, 1 H triazolic), 8.93 (d, J = 3 Hz 1 H CH Ar). ^{13}C NMR (75 MHz, DMSOd$_6$): 14.33 (CH$_3$), 22.37, 26.22, 28.71, 28.92, 29.94, 31.54, 45.25, 49.86 (CH$_2$), 108.60, 116.66, 121.09, 124.11 (CH Ar), 124.21, 124.21, 132.81, 139.81, 142.30, 147.53 (C$_q$). HRMS (ESI) Calculated for $C_{19}H_{25}N_6O_2Cl$: [M+H$^+$] = 404.1724, Found: [M+H$^+$] = 404.1702. Elemental analysis Calculated: C, 56.36%; H, 6.22%; N, 20.76%; O, 7.90%; Cl, 8.76 % Found: C, 56.47%; H, 6.11%; N, 20.66%; O, 7.94%; Cl, 8.82 %.

3-chloro-6-nitro-1-((1-nonyl-1H-1,2,3-triazol-5-yl)methyl)-1H-indazole (10a)

Brown solid, Yield = 19 %. m.p: 149-151 °C. ^{1}H NMR (300 MHz, DMSOd$_6$): 0.86 (t, J = 9 Hz 3 H CH$_3$), 1.24-1.32 (m, 12 H CH$_2$), 2.0 (t, J = 5.1 Hz 12 H CH$_2$), 4.27 (t, J = 9 Hz 2 H CH$_2$), 5.28 (s, 2H CH$_2$), 7.25 (s, 1 H triazolic), 7.78 (d, J = 9 Hz 1 H CH Ar), 7.90 (m, 1 H CH Ar), 8.68 (d, J = 1.5 Hz 1 H CH Ar). ^{13}C NMR (75 MHz, DMSOd$_6$): 14.58 (CH$_3$), 22.37-35.64 (6 CH$_2$), 46.54-52.57 (CH$_2$), 107.30-115.58-122.85 (CH Ar), 125.29 (CH triazolic), 128.19-129.09-134.52-137.49-138.54-146.58 (C$_q$). HRMS (ESI) Calculated for $C_{19}H_{25}N_6O_2Cl$: [M+H$^+$] = 404.1724, Found: [M+H$^+$] = 404.1717. Elemental analysis Calculated: C, 56.36%; H, 6.22%; N, 20.76%; O, 7.90%; Cl, 8.76 % Found: C, 56.43%; H, 6.15%; N, 20.68%; O, 7.84%; Cl, 8.90 %.

1-((1-benzyl-1H-1,2,3-triazol-4-yl)methyl)-3-chloro-6-nitro-1H-indazole (11)

Brown solid, Yield = 66 %. m.p: 160-162 °C. ^{1}H NMR (300 MHz, DMSOd$_6$): 5.53 (s, 2 H CH$_2$), 5.77 (s, 2 H CH$_2$), 8.23 (s, 1 H CH triazolic), 7.25-7.31 (m, 5H CH Ar), 7.76 (d, J = 9 Hz 1 H CH Ar), 8.01 (d, J = 9 Hz 1 H CH Ar), 8.99 (d, J = 1.5 Hz 1 H CH Ar). ^{13}C NMR (75 MHz, DMSOd$_6$): 45.09 (CH$_2$), 53.35 (CH$_2$), 108.42, 116.69, 120.76, 128.42, 128.64, 129.91 (CH Ar), 126.44 (Cq triazolic), 122.02, 125.02, 129.20, 136.28, 139.71, 147.53 (C$_q$). HRMS

(ESI) Calculated for $C_{17}H_{13}N_6O_2Cl$: [M+H$^+$] = 368.0814, Found: [M+H$^+$] = 368.0831. Elemental analysis Calculated: C, 55.37%; H, 3.55%; N, 22.79%; O, 8.68%; Cl, 9.61 % Found: C, 55.29%; H, 3.46%; N, 22.93%; O, 8.76%; Cl, 9.56 %.

General procedure for the synthesis of compounds 10, 11, 12 and 13

One millimole of 3-chloro-6-nitro-1-(prop-2-yn-1-yl)-1H-indazole **3** and 1.5 mmol of azide were dissolved in 25 mL of ethanol at room temperature in a 100 mL flask, followed by the addition of 0.5 mmol of $CuSO_4$ and 1 mmol of sodium ascorbate dissolved in 10 mL of distilled water. The reaction mixture was stirred for 24 h and proceeded by TLC. After filtration, the residue obtained was purified by chromatography on a silica column. Eluent: a mixture of hexane and ethyl acetate (90/10).

1-((1-benzyl-1H-1,2,3-triazol-4-yl)methyl)-3-chloro-6-nitro-1H-indazole (11)

Brown solid, Yield = 75 %. m.p: 160-162 ˚C. ^{1}H NMR (300 MHz, DMSOd$_6$): 5.53 (s, 2 H CH_2), 5.77 (s, 2 H CH_2), 8.23 (s, 1 H CH triazolic), 7.25-7.31 (m, 5H CH Ar), 7.76 (d, J = 9 Hz 1 H CH Ar), 8.01 (d, J = 9 Hz 1 H CH Ar), 8.99 (d, J = 1.5 Hz 1 H CH Ar). ^{13}C NMR (75 MHz, DMSOd$_6$): 45.09 (CH_2), 53.35 (CH_2), 108.42, 116.69, 120.76, 128.42, 129.20 (CH Ar), 126.44 (C_q triazolic), 122.02, 125.02, 129.20, 136.28, 139.71, 147.53 (C_q). HRMS (ESI) Calculated for $C_{17}H_{13}N_6O_2Cl$: [M+H$^+$] = 368.0814, Found: [M+H$^+$] = 368.0833. Elemental analysis Calculated: C, 55.37%; H, 3.55%; N, 22.79%; O, 8.68%; Cl, 9.61 % Found: C, 55.23%; H, 3.47%; N, 22.66%; O, 8.79%; Cl, 9.85 %.

3-chloro-6-nitro-1-((1-octyl-1H-1,2,3-triazol-4-yl) methyl)-1H-indazole (12)

Brown solid, Yield = 63 %. m.p: 166-168 ˚C. ^{1}H NMR (300 MHz, DMSOd$_6$): 0.77 (t, J = 6 Hz 3 H CH_3), 1.16-1.28 (m, 12 H CH_2), 1.71-1.78 (m, 2 H CH_2), 4.29 (t, J = 6 Hz, 2 H CH_2), 5.86 (s, 2 H CH_2), 8.15 (s, 1 H CH triazolic), 7.90 (d, J = 6 Hz 1 H CH Ar), 8.02 (d, J = 3 Hz 1 H CH Ar), 8.92 (d, J = 3 Hz 1 H CH Ar). ^{13}C NMR (75 MHz, DMSOd$_6$): 14.33 (CH_3), 22.37, 49.86 (7 CH_2), 108.60, 116.66, 121.09, 124.21 (CH Ar), 124.11 (CH triazolic), 124.11, 132.81, 139.81, 142.30, 147.53 (C_q). HRMS (ESI) Calculated for $C_{18}H_{23}N_6O_2Cl$: [M+H$^+$] = 390.1678, Found: [M+H$^+$] = 390.1696. Elemental analysis Calculated: C, 55.31%; H, 5.93%; N, 21.50%; O, 8.19%; Cl, 9.07 % Found: C, 55.46%; H, 5.79%; N, 21.47%; O, 8.32%; Cl, 8.96 %.

Ethyl-2-(4-((3-chloro-6-nitro-1H-indazol-1-yl)methyl)-1H-1,2,3-triazol-1-yl)acetate (13)

Brown solid, Yield = 77 %. m.p: 142-144 ˚C. ^{1}H NMR (300 MHz, DMSOd$_6$): 1.28 (t, J = 7.2

Hz 3 H CH_3), 4.24 (t, J = 7.2 Hz 2 H CH_2), 5.24 (s, 2 H CH_2), 5.84 (s, 2 H CH_2), 8.46 (s, 1 H CH triazolic), 7.90 (dd, J = 6 Hz, J = 9 Hz 1 H CH Ar), 8.01 (dd, J = 1.8 Hz, J = 8.7 Hz 1 H CH Ar), 8.93 (d, J = 3 Hz, 1 H CH Ar). ^{13}C NMR (75 MHz, DMSOd$_6$): 14.09 (CH_3), 47.09, 50.67, 62.24 (3 CH_2), 105.78, 115.92, 122.11 (CH Ar), 124.21 (CH triazolic), 127.52, 131.11-134.61, 139.09, 146.87, 167.21 (C_q). HRMS (ESI) Calculated for $C_{14}H_{13}N_6O_4Cl$: [M+H$^+$] = 364.0738, Found: [M+H$^+$] = 364.0754. Elemental analysis Calculated: C, 46.10%; H, 3.59%; N, 23.04%; O, 17.55%; Cl, 9.72 % Found: C, 46.21%; H, 3.53%; N, 22.94%; O, 17.65%; Cl, 9.67 %.

General procedure for the synthesis of compounds 14, and 15

To a solution of 0.01 mol of 3-chloro-6-nitro-1-(prop-2-yn-1-yl)-1H-indazole **3** and 0.02 mol of benzaldehyde oxime in 40 mL of dichloromethane with magnetic stirring at 0 ˚C, 20 ml of a bleach solution was added dropwise. After 5 hours of stirring, the organic phases were decanted, washed twice with 20 ml of water and dried over magnesium sulfate. After filtration and removal of the solvent under reduced pressure, the products were purified by chromatography on a silica column with a mixture of hexane and ethyl acetate (90/10) as eluent.

5-((3-chloro-6-nitro-1H-indazol-1-yl) methyl)-3-phenylisoxazole (14)

Brown solid, Yield = 68%. m.p: 171-173 ˚C. ^{1}H NMR (300 MHz, DMSOd$_6$): 6.16 (s, 2 H CH_2), 7.05 (s, 1 H CH), 7.46-7.49 (m, 1 H CH Ar), 7.81 (d, J = 9 Hz, 1 H CH Ar), 8.08 (d, J = 9 Hz 1H CH Ar), 9.04 (d, J = 3 Hz 1 H). ^{13}C NMR (75 MHz, DMSOd$_6$): 45.08 (CH_2), 102.27 (CH), 108.24, 117.14, 121.34, 121.92, 127.13, 129.59, 130.88 (CH Ar), 126.66, 128.54, 140.26, 147.99, 162.60, 168.16 (C_q). HRMS (ESI) Calculated for $C_{17}H_{11}N_4O_3Cl$: [M+H$^+$] = 354.0518, Found: [M+H$^+$] = 354.0539. Elemental analysis Calculated: C, 57.56%; H, 3.13%; N, 15.79%; O, 13.53%; Cl, 9.99 % Found: C, 57.71%; H, 3.04%; N, 15.67%; O, 13.43%; Cl, 10.15 %.

5-((3-chloro-6-nitro-1H-indazol-1-yl) methyl)-3-(4-chlorophenyl)isoxazole (15)

Brown solid, Yield = 63%. m.p: 191-193 ˚C. ^{1}H NMR (300 MHz, DMSOd$_6$): 6.16 (s, 2 H CH_2), 7.02 (s, 1 H CH), 7.40-7.60 (m, 1 H CH Ar), 7.78 (d, J = 9 Hz, 1 H CH Ar), 8.02 (d, J = 12 Hz 1H CH Ar), 9.04 (s, 1 H). ^{13}C NMR (75 MHz, DMSOd$_6$): 44.92 (CH_2), 105.44 (CH), 108.19, 117.03, 121.76, 126.60, 128.09, 130.81 (CH Ar), 123.92, 131.48, 132.12, 140.19, 147.90, 161.21, 167.34 (C_q). HRMS (ESI) Calculated for $C_{17}H_{10}N_4O_3Cl_2$: [M+H$^+$] = 388.0110, Found: [M+H$^+$] = 388.0129. Elemental analysis Calculated: C, 52.46%; H, 2.59%; N, 14.40%; O, 12.33%; Cl, 18.22 % Found: C, 52.33%; H, 2.51%; N, 14.32%; O, 12.47%; Cl,

18.37 %.

NMR spectra

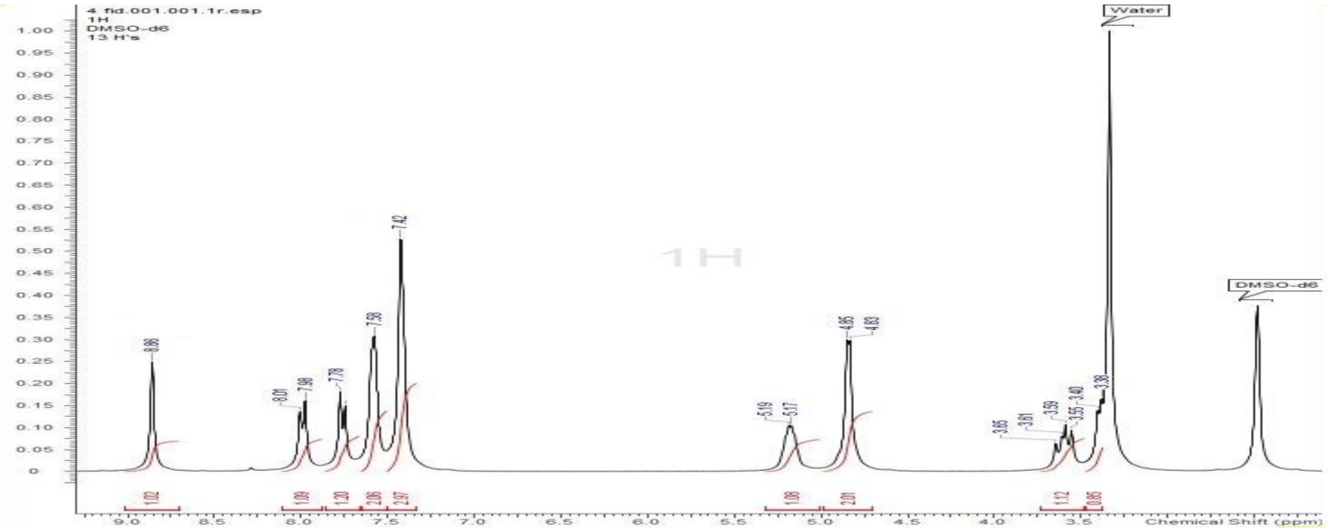

Figure S1. The ^{1}H-NMR of **4**

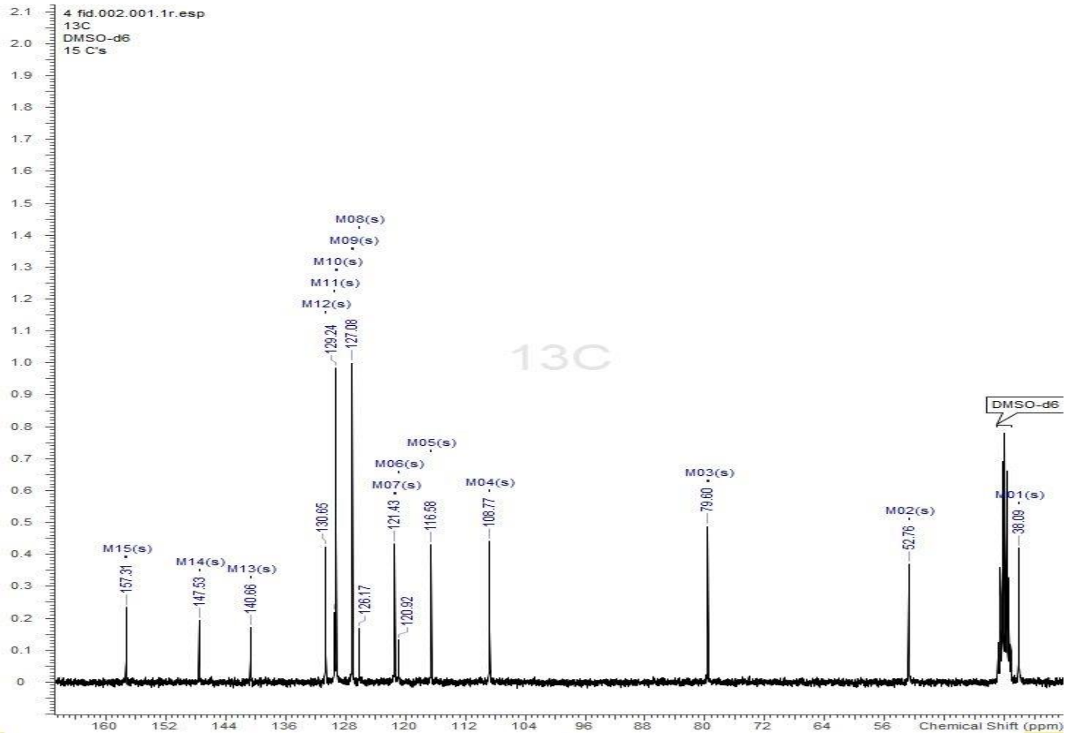

Figure S2. The ^{13}C-NMR of **4**

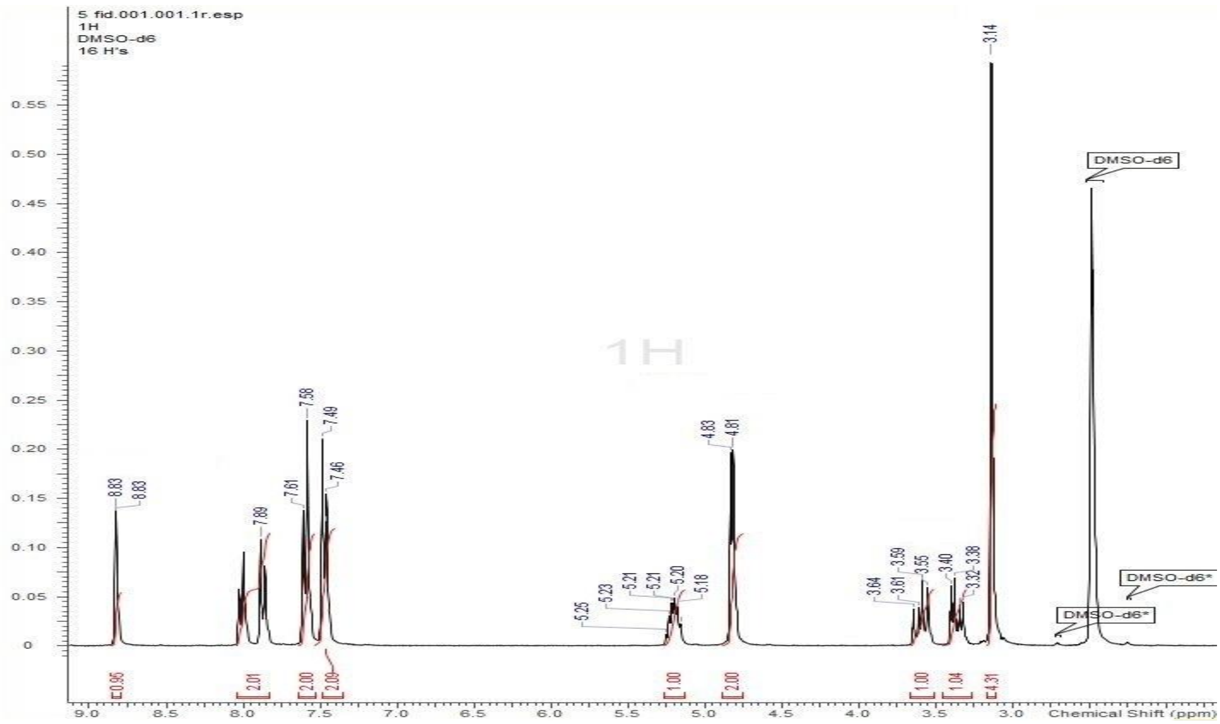

Figure S3. The ^{1}H-NMR of **5**

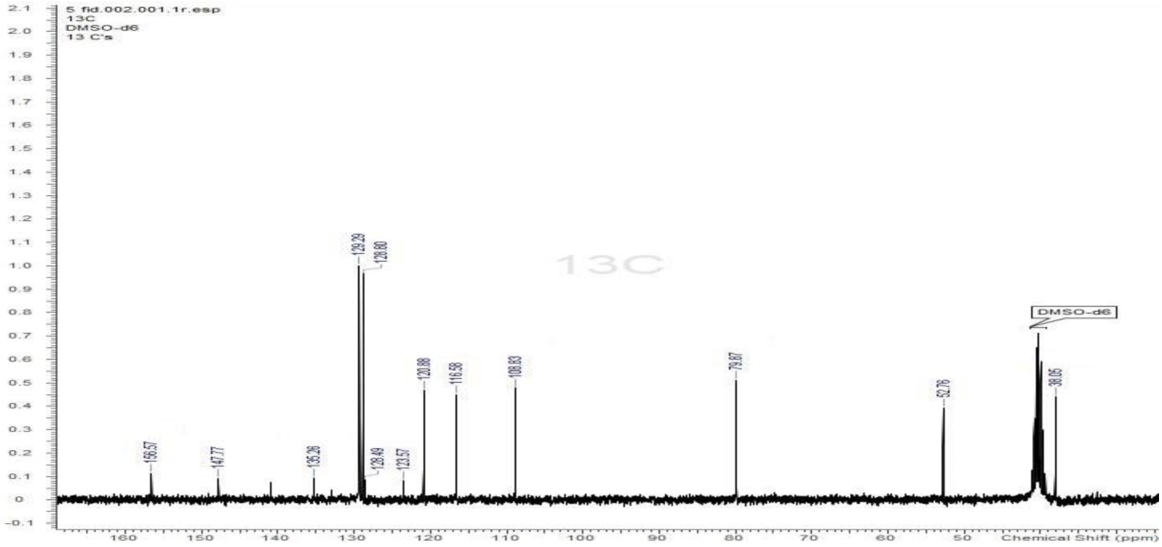

Figure S4. The ^{13}C-NMR of **5**

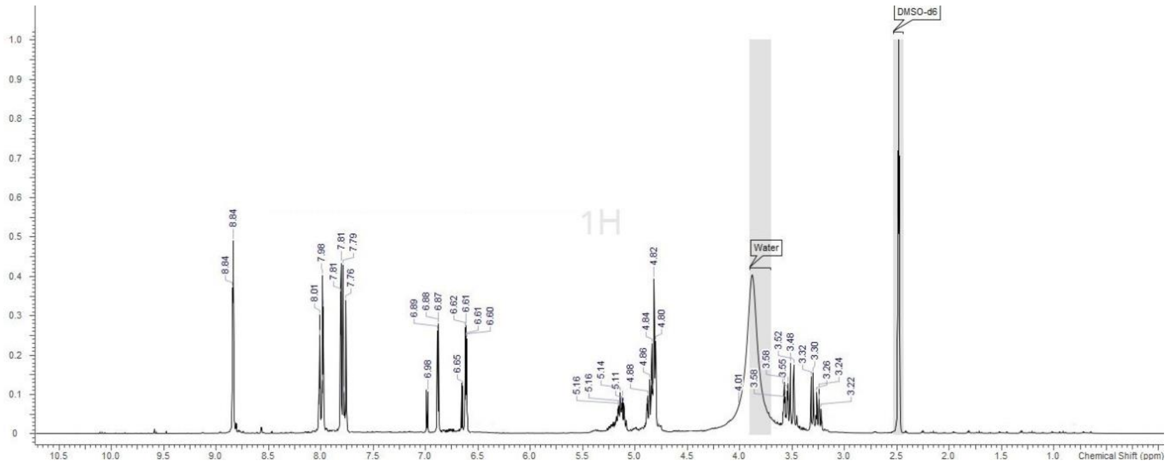

Figure S5. The ^{1}H-NMR of **6**

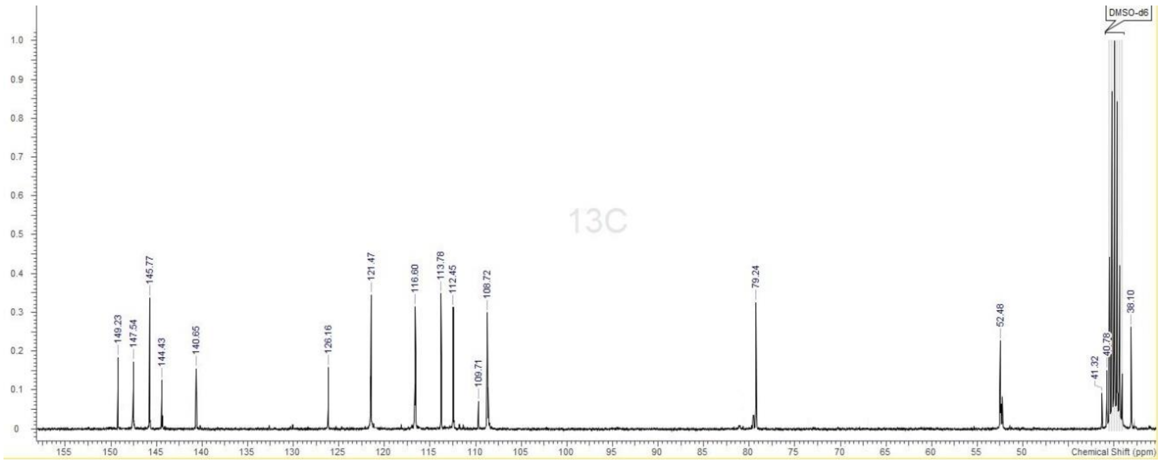

Figure S6. The ^{13}C-NMR of **6**

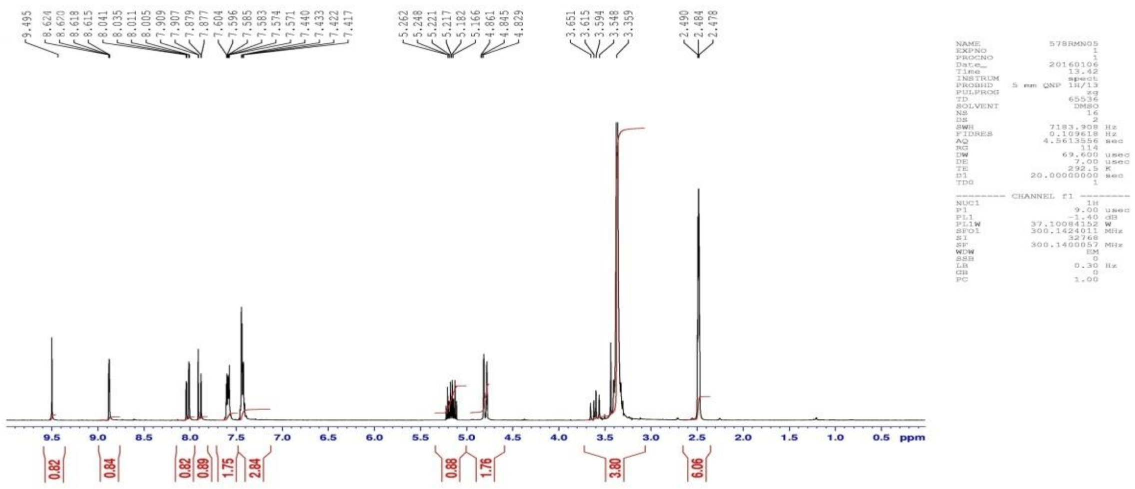

Figure S7. The ^{1}H-NMR of **7**

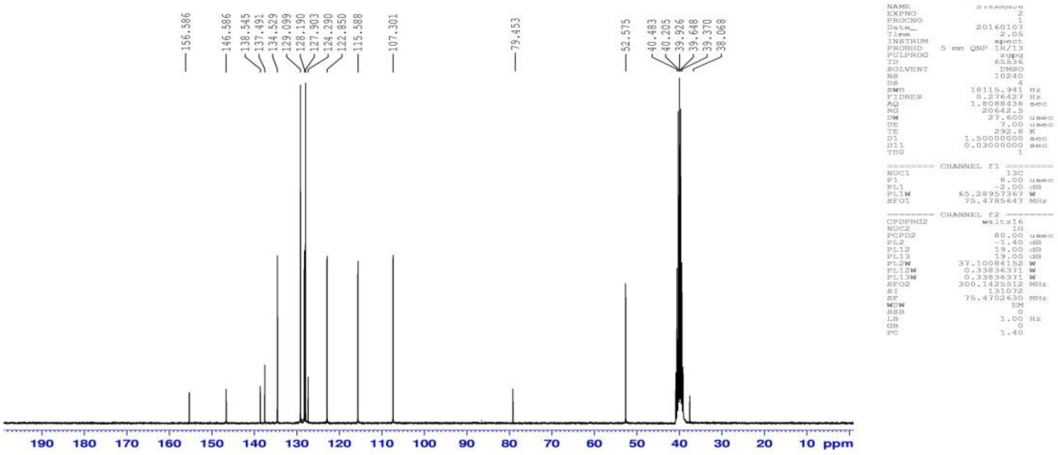

Figure S8. The ^{13}C-NMR of **7**

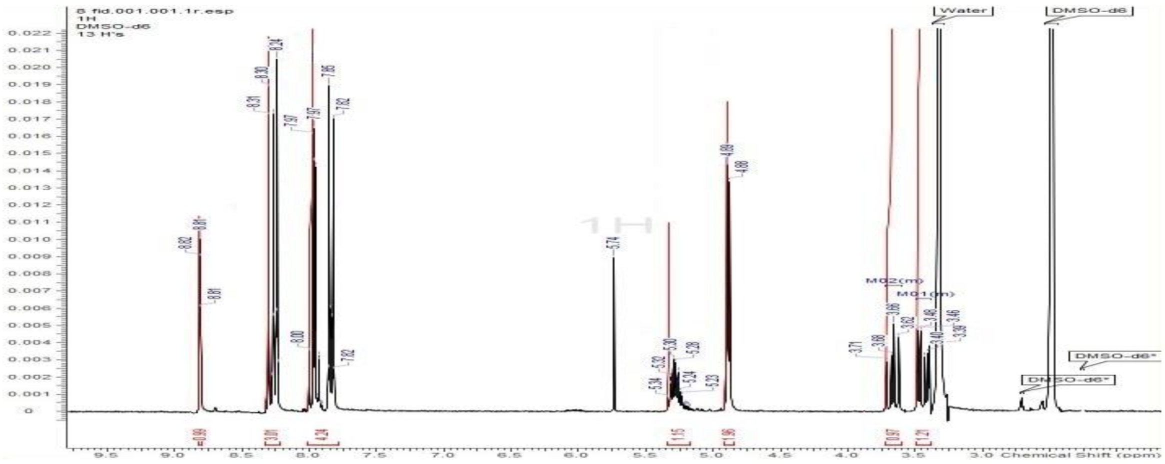

Figure S9. The ^{1}H-NMR of **8**

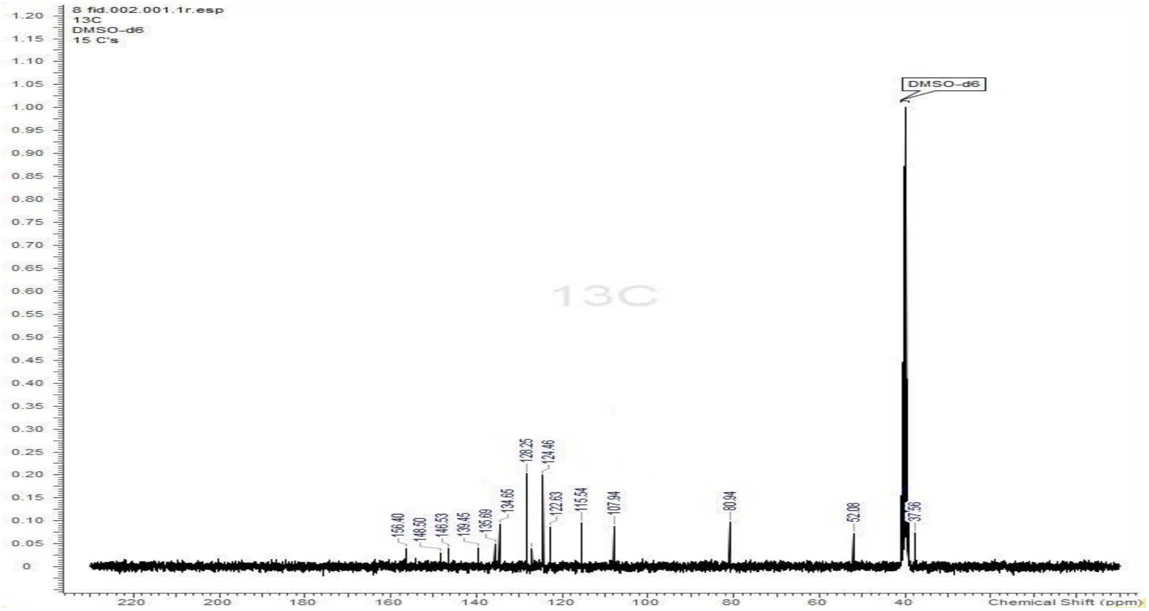

Figure S10. The ^{13}C-NMR of **8**

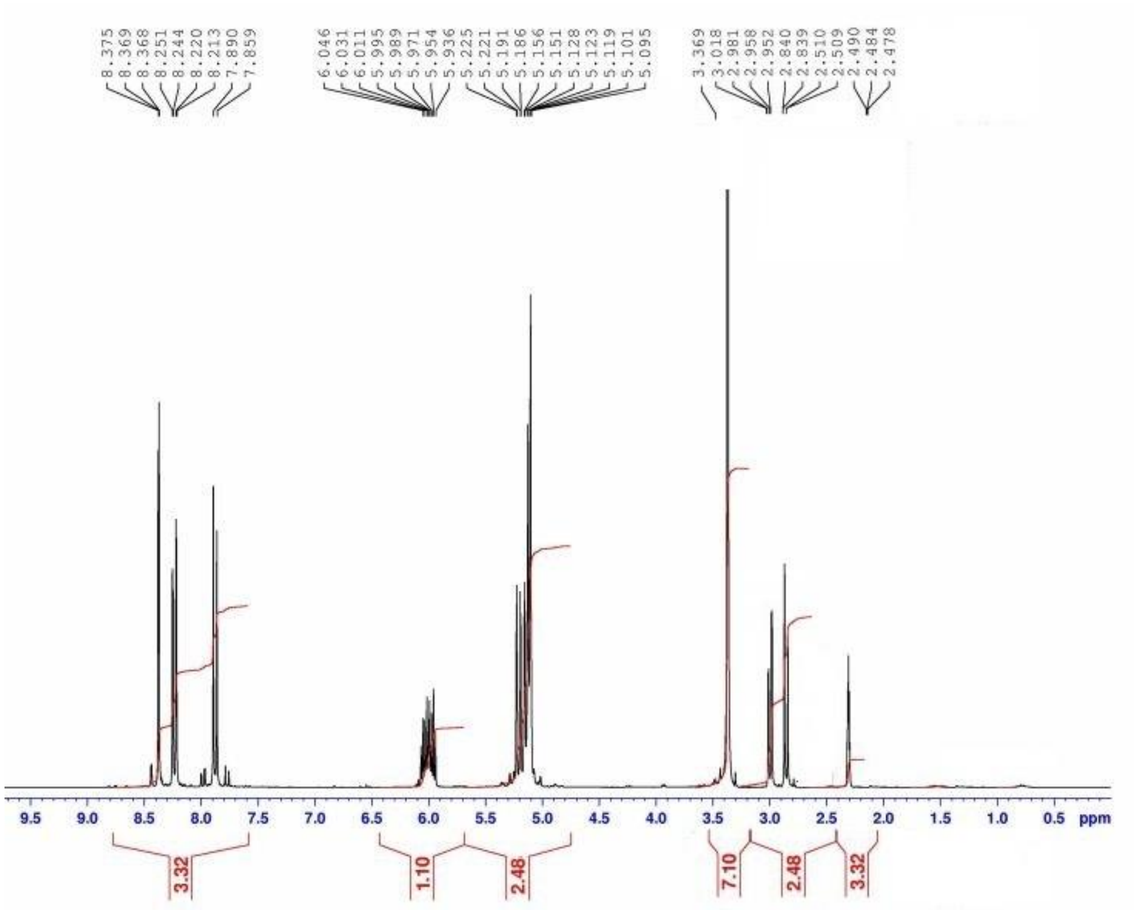

Figure S11. The ^{1}H-NMR of **9**

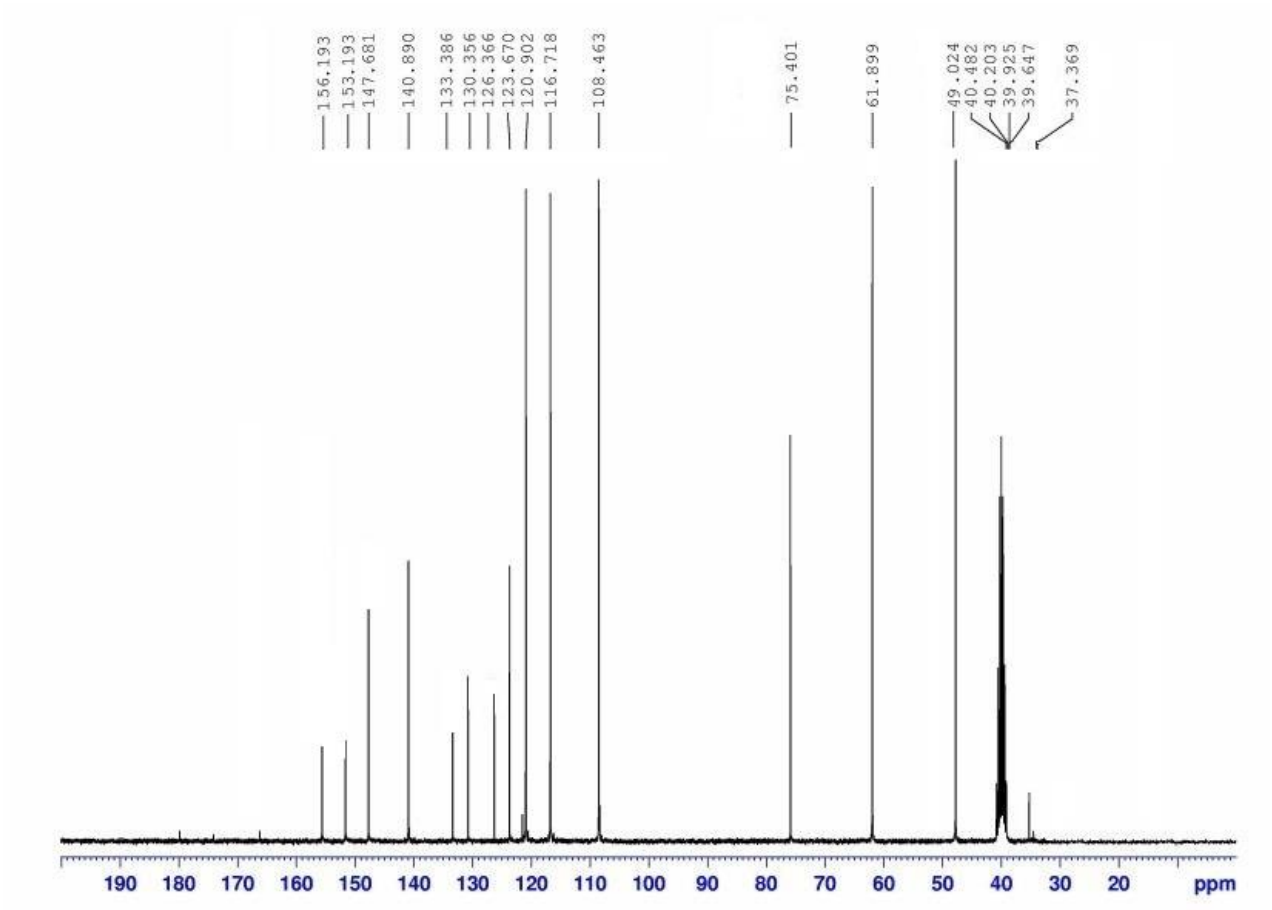

Figure S12. The ^{13}C-NMR of **9**

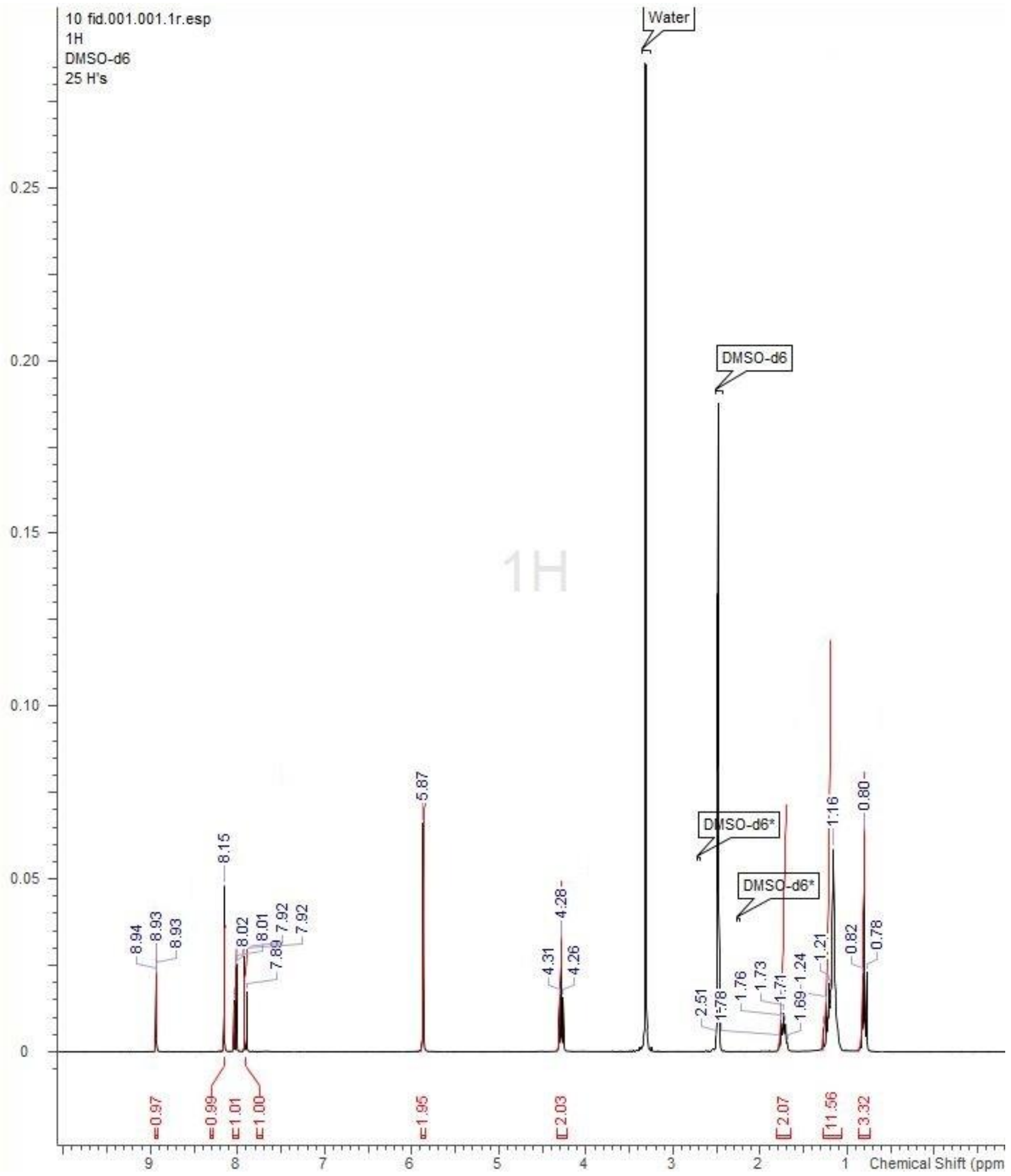

Figure S13. The ^{1}H-NMR of **10**

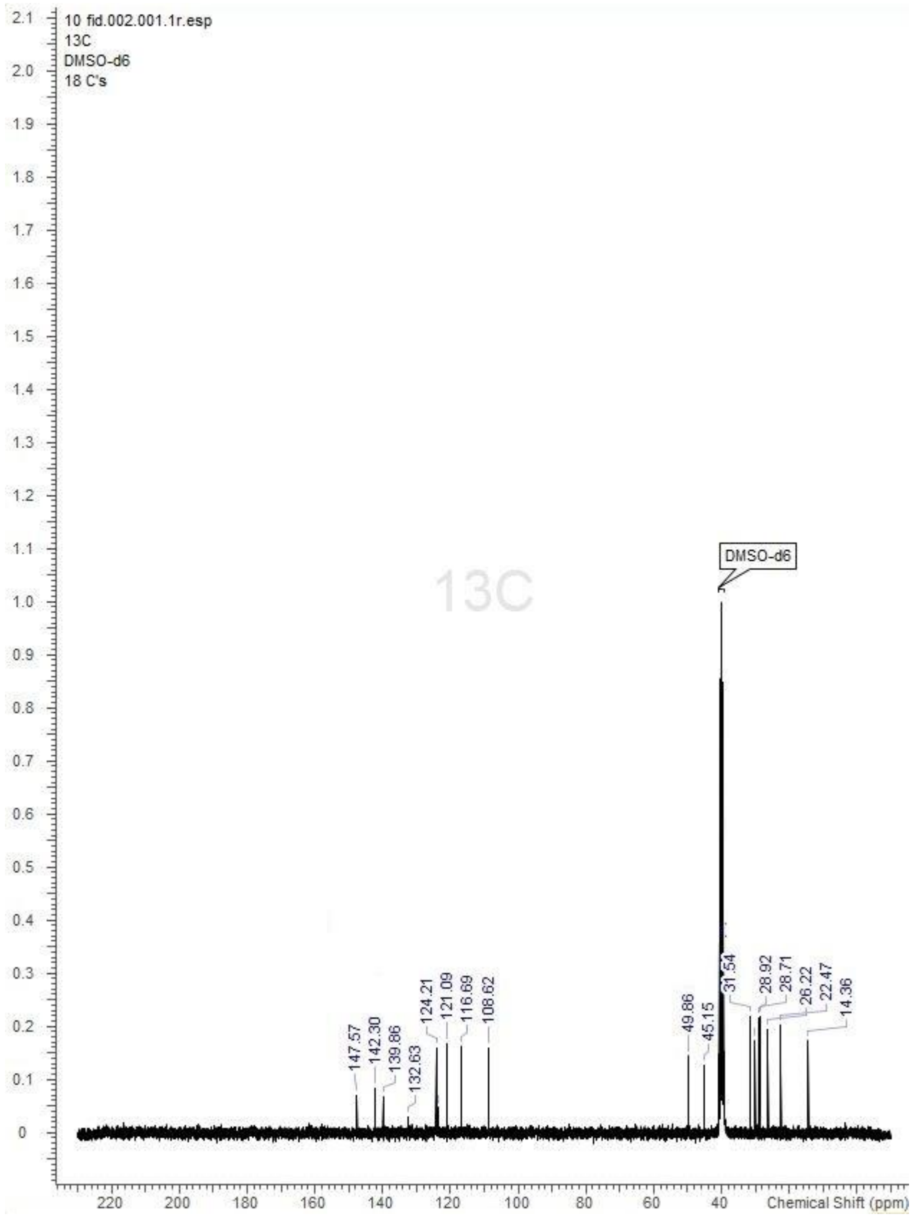

Figure S14. The ^{13}C-NMR of **10**

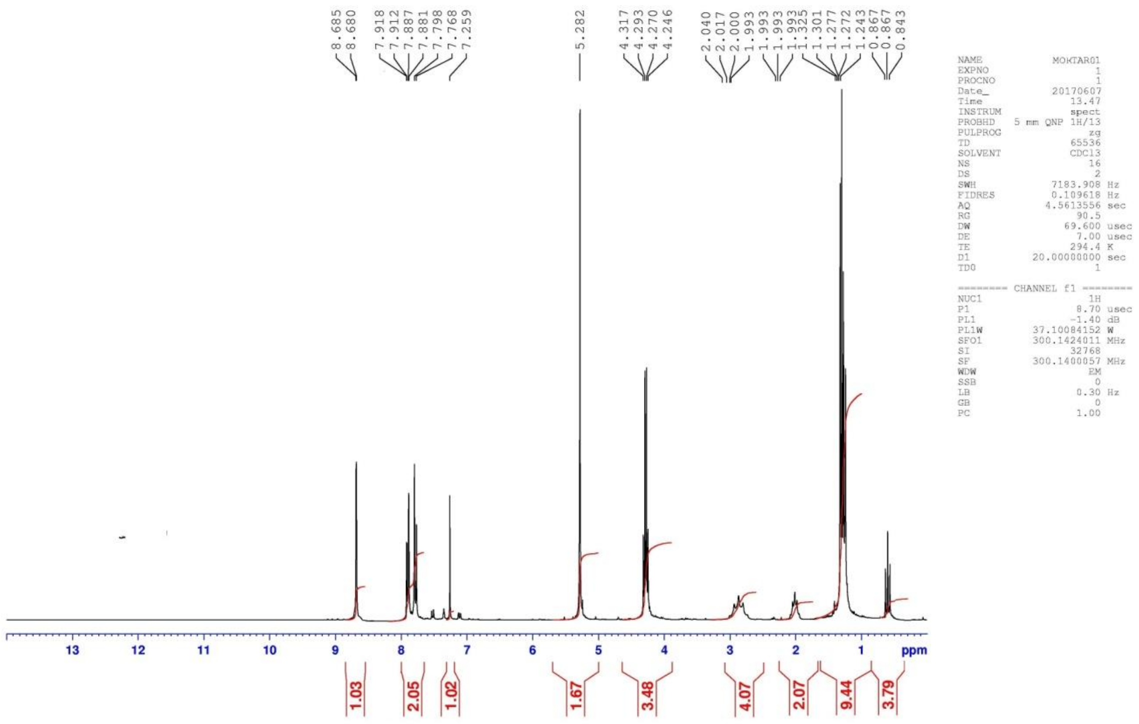

Figure S15. The ^{1}H-NMR of **10a**

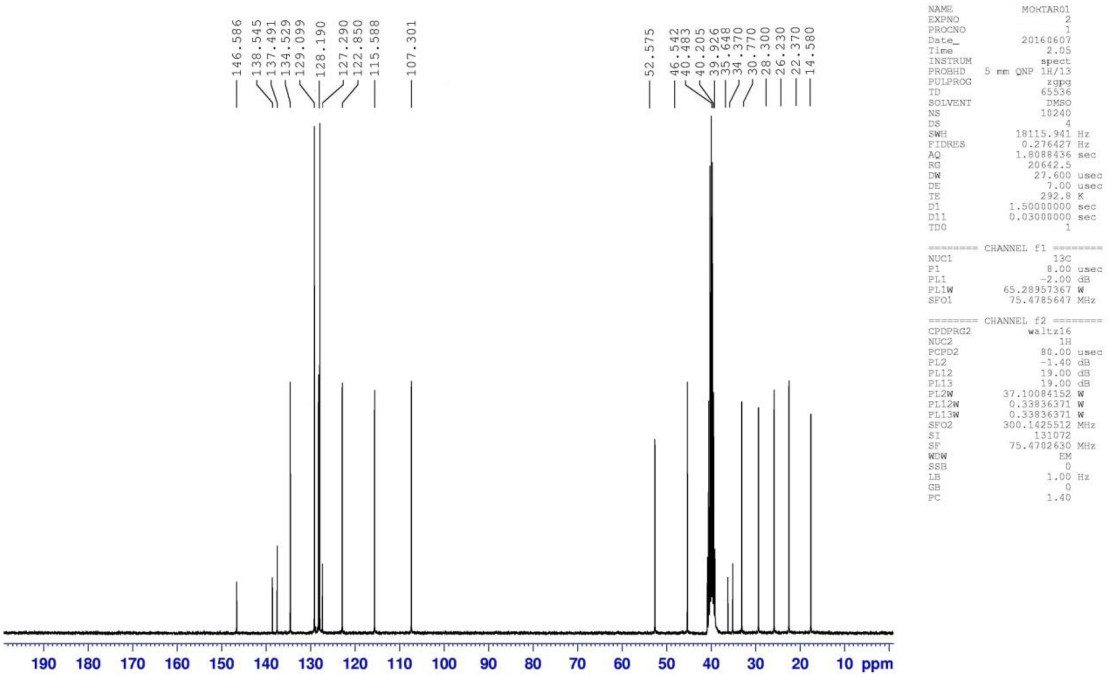

Figure S16. The ^{13}C-NMR of **10a**

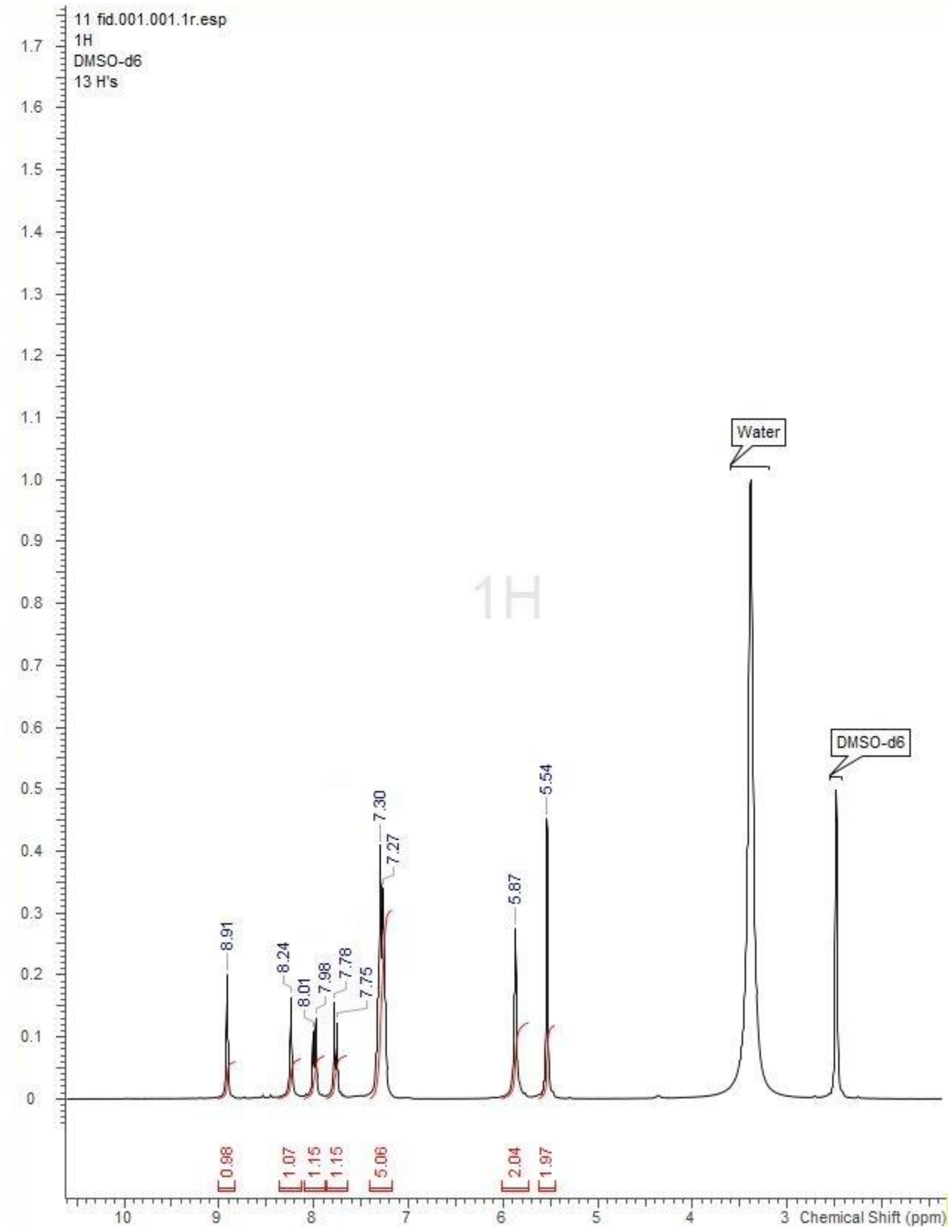

Figure S17. The ^{1}H-NMR of **11**

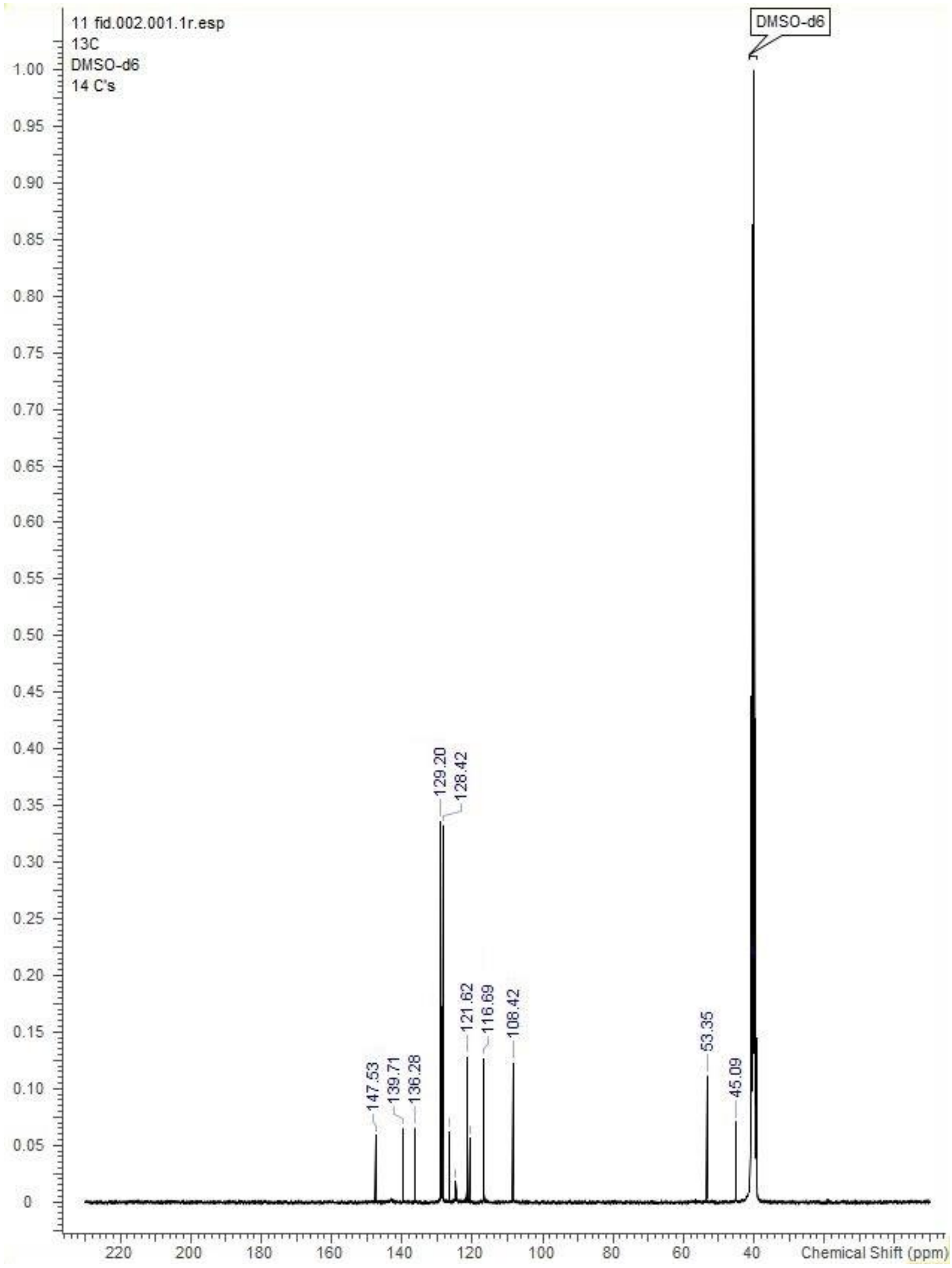

Figure S18. The ^{13}C-NMR of **11**

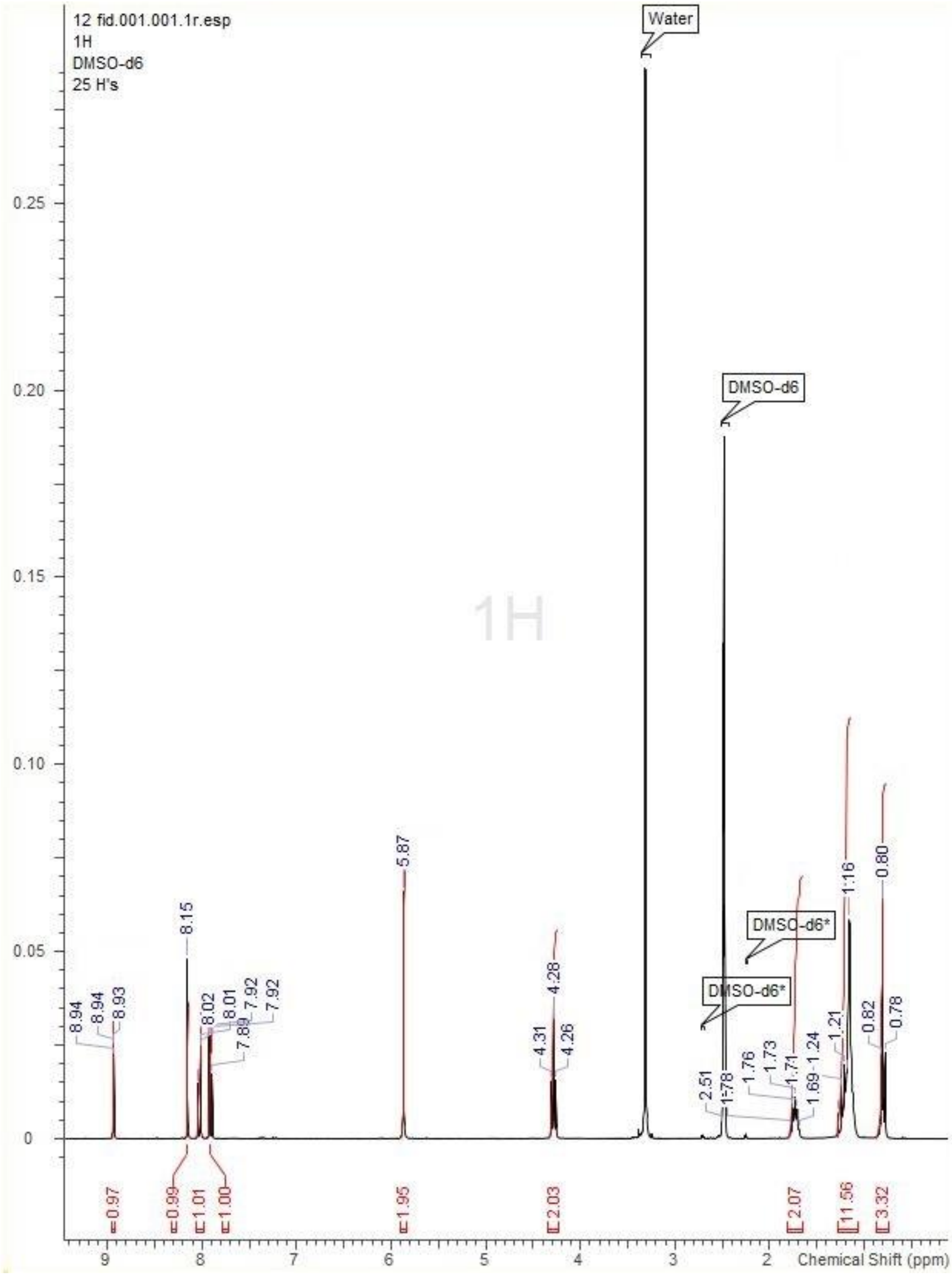

Figure S19. The ^{1}H-NMR of **12**

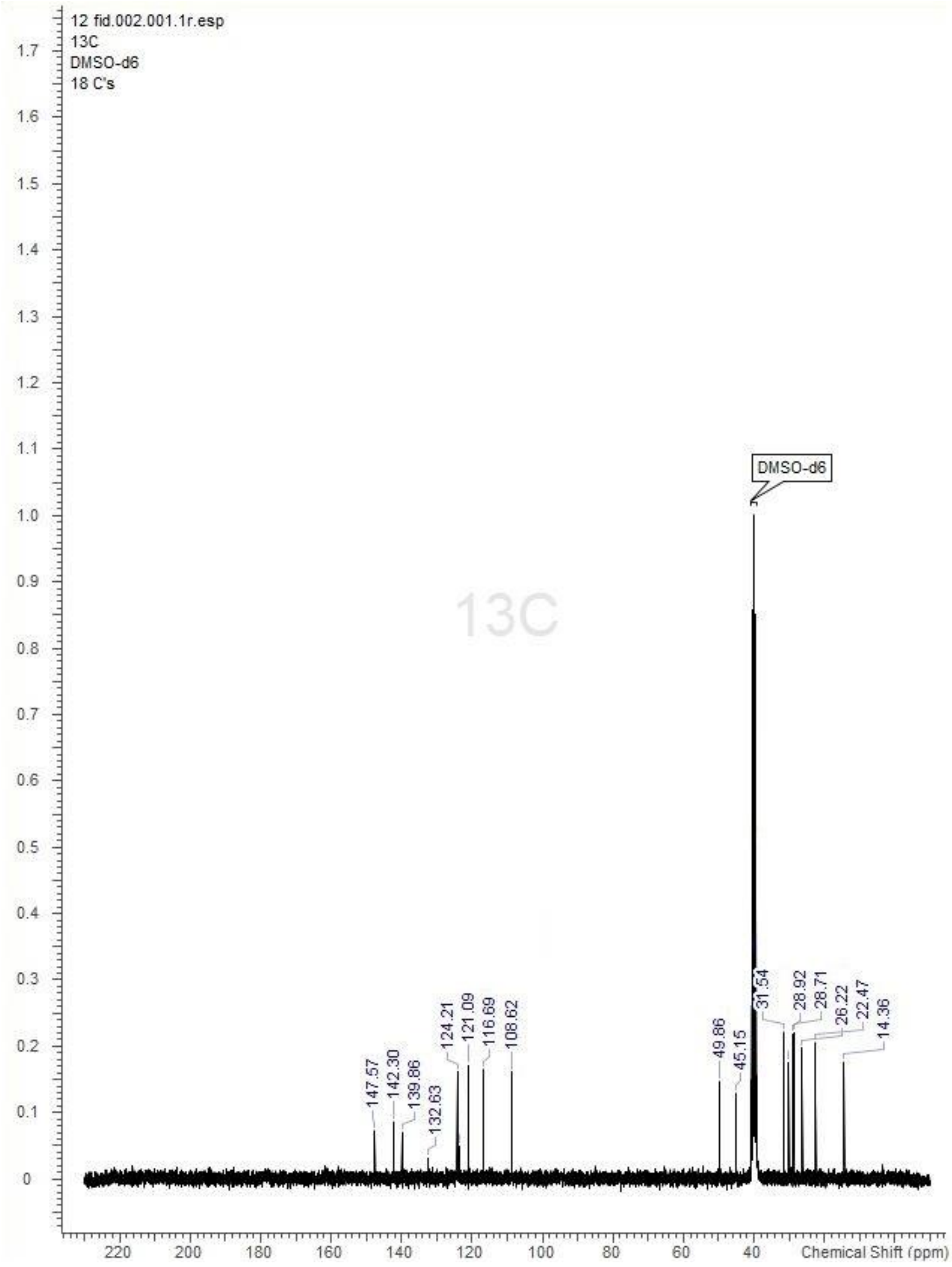

Figure S20. The ^{13}C-NMR of **12**

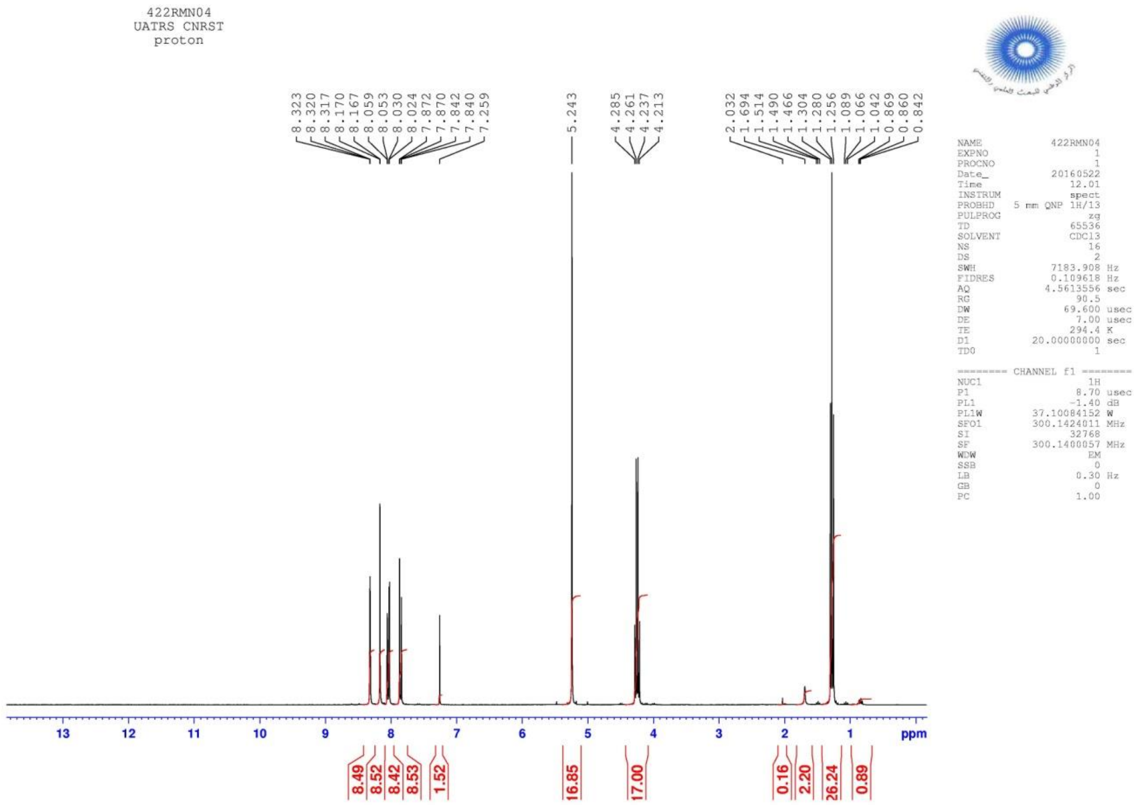

Figure S21. The ^{1}H-NMR of **13**

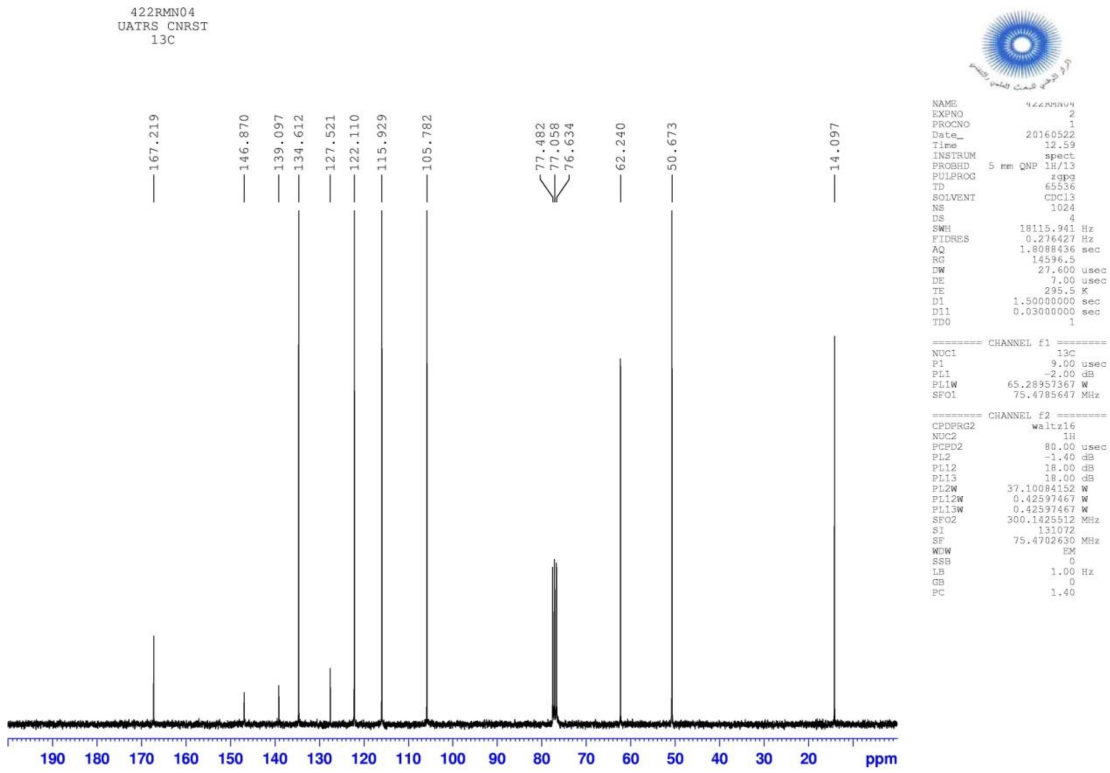

Figure S22. The ^{13}C-NMR of **13**

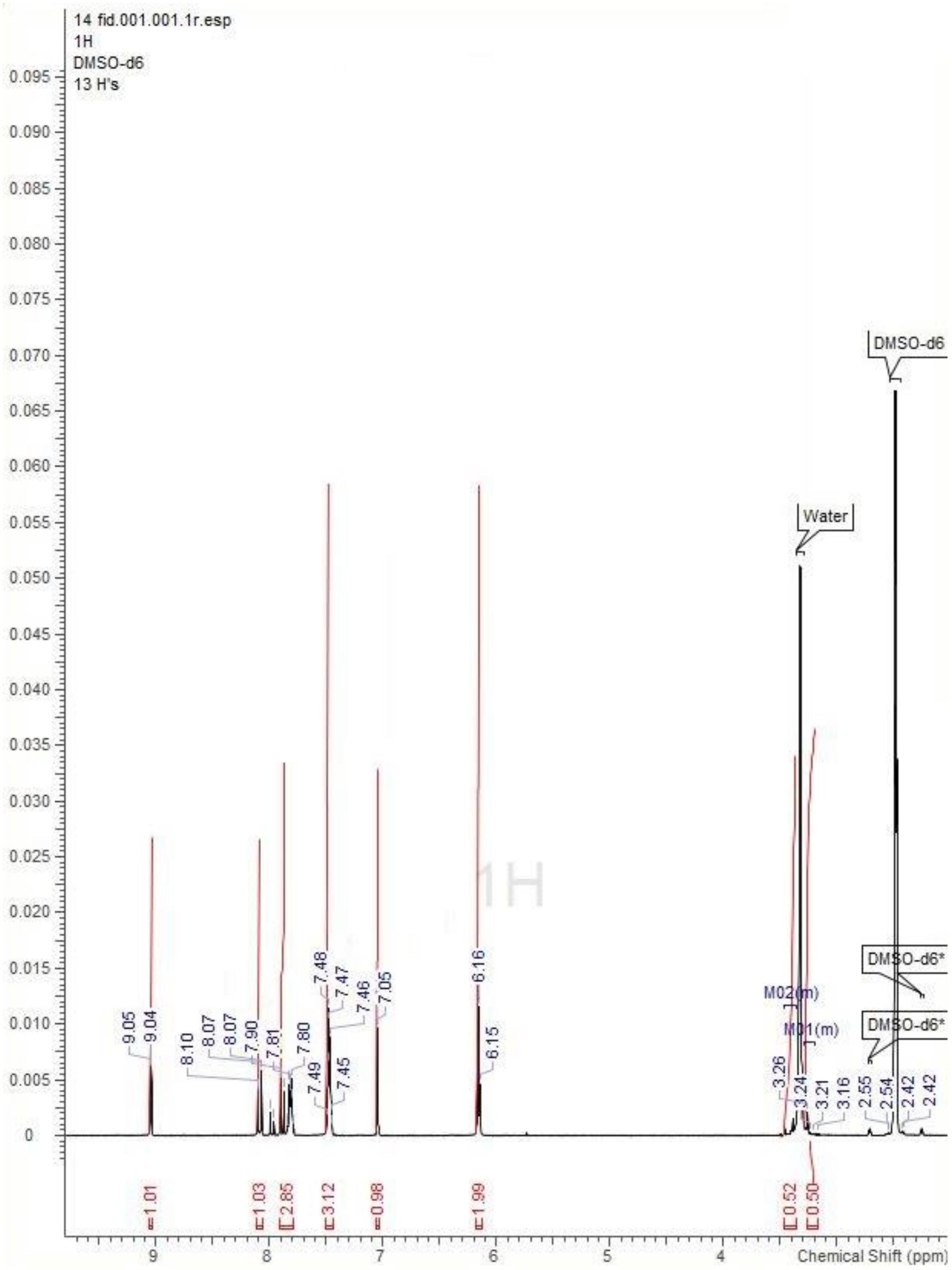

Figure S23. The ^{1}H-NMR of **14**

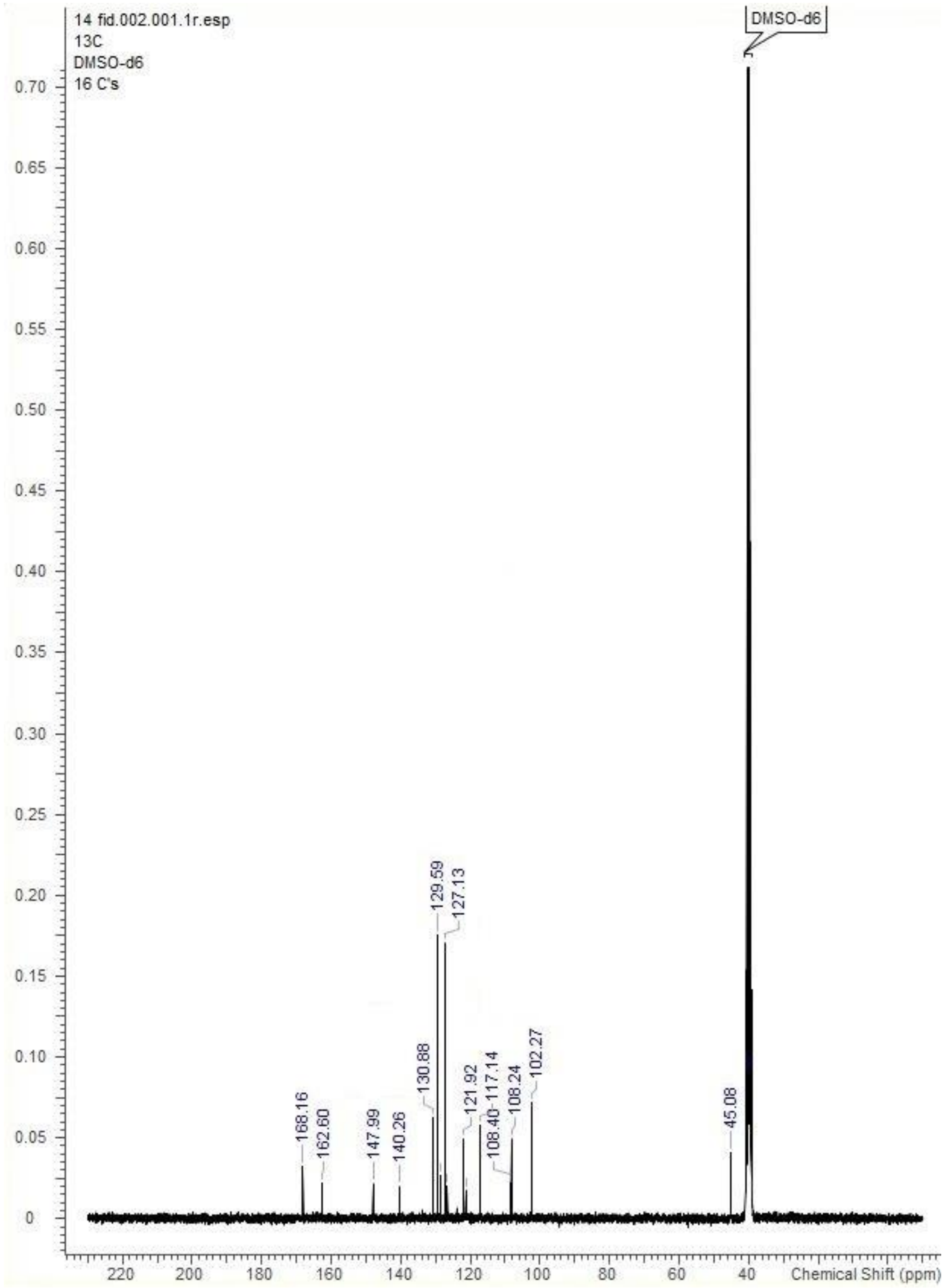

Figure S24. The ^{13}C-NMR of **14**

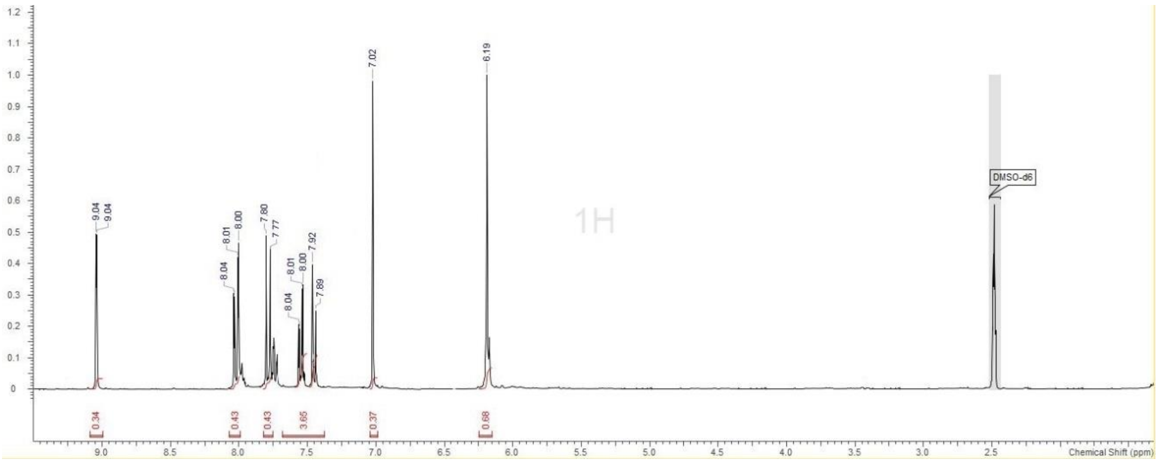

Figure S25. The ^{1}H-NMR of **15**

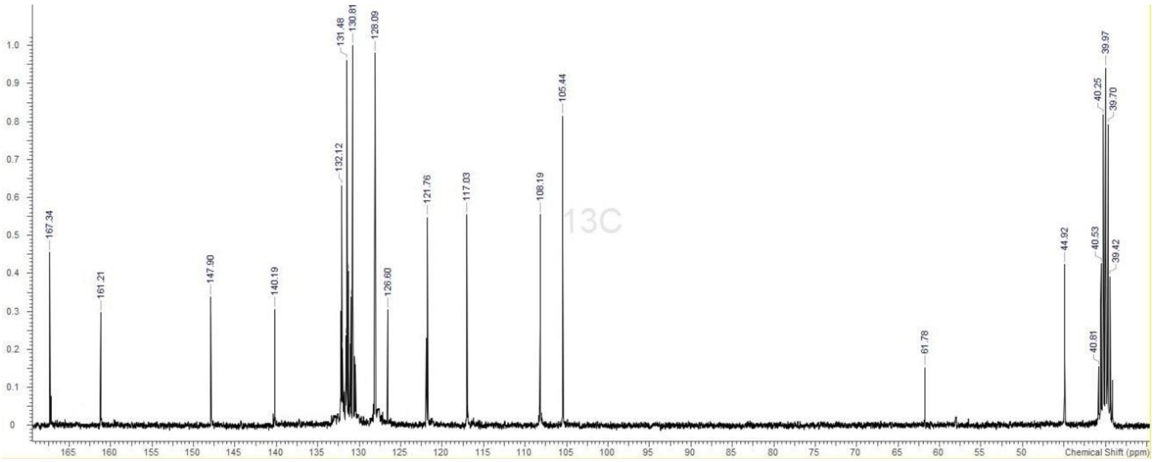

Figure S26. The ^{13}C-NMR of **15**

The converged geometry of the tested compounds in this study

The converged geometry of 4

C	-4.882951955	0.637149845	0.378168406
C	-4.417445106	-0.675184539	0.131001846
C	-3.111822611	-0.980072868	-0.224162755
C	-2.236686639	0.111713390	-0.331804595
C	-2.671943197	1.443114079	-0.088549560
C	-4.008518640	1.705604860	0.268012000
N	-0.907623963	0.189317657	-0.667537203
N	-0.467487596	1.468371773	-0.643402222
C	-1.492345921	2.219920557	-0.307028715
N	-5.376779682	-1.797333667	0.257303083
O	-4.957881389	-2.924112503	0.046354585
O	-6.524880260	-1.524312656	0.564388306
Cl	-1.360661697	3.931864876	-0.171832812
C	0.824768151	-1.356036645	0.273792966
O	1.553833793	-2.536688937	-0.132743461
N	2.897333996	-2.235587384	-0.271014004
C	3.141738832	-1.055702002	0.179785364
C	1.907910031	-0.374399645	0.736933597
C	4.499044868	-0.489739831	0.174127549
C	0.014425230	-0.893631886	-0.950722555
C	5.584028406	-1.230631591	-0.338135339
C	6.868168284	-0.692629015	-0.337402818
C	7.097705160	0.592059202	0.173250041
C	6.030398835	1.334313623	0.683001228
C	4.739351464	0.798804424	0.683322735
H	-5.927989755	0.774624368	0.651629658
H	-2.822614724	-2.015219446	-0.399530697
H	-4.341610789	2.728733113	0.452191553
H	0.138302049	-1.653753840	1.081308688
H	1.973724012	-0.298018059	1.835249391
H	1.753514806	0.640453567	0.341929910

H	0.696657832	-0.563349505	-1.747442608
H	-0.560399104	-1.748413405	-1.333916391
H	5.397993287	-2.230910659	-0.732851831
H	7.700011477	-1.277927813	-0.737499875
H	8.107035302	1.011198196	0.172044111
H	6.200209592	2.337328891	1.082266134
H	3.914787263	1.393174167	1.082800344

HF= -1559.42158518 hartree

Imaginary Freq 0

The converged geometry of 5

C	5.540859237	0.809763066	-0.405465965
C	5.146911938	-0.525639333	-0.157850417
C	3.863268667	-0.899016604	0.211721200
C	2.934107783	0.145627334	0.333778261
C	3.297165032	1.498597123	0.091150739
C	4.613882506	1.831223749	-0.280251766
N	1.606558109	0.153404528	0.684594152
N	1.100876451	1.408469099	0.670839782
C	2.081725945	2.212958448	0.326220668
N	6.161676333	-1.596104323	-0.300223442
O	5.805032617	-2.743488390	-0.086127362
O	7.289365857	-1.262826846	-0.622682715
Cl	1.860054617	3.916075619	0.199711032
C	-0.055361413	-1.471758777	-0.246474632
O	-0.731961151	-2.682580703	0.164435869
N	-2.085852443	-2.441519616	0.303376091
C	-2.382745259	-1.273181040	-0.146484665
C	-1.181656829	-0.536131384	-0.703140465
C	-3.763186908	-0.770613570	-0.139467271
C	0.743034825	-0.975317803	0.972519731
C	-4.816130299	-1.563452547	0.361688636
C	-6.124780349	-1.093052730	0.365609028
C	-6.401357749	0.186077148	-0.135723909

C	-5.376220818	0.990443404	-0.637333099
C	-4.065449386	0.509198211	-0.636167951
H	6.573985369	1.002033809	-0.690963678
H	3.630449934	-1.948412036	0.386541264
H	4.891462427	2.870874679	-0.463956048
H	0.637197021	-1.741017994	-1.058699813
H	-1.252734796	-0.457186092	-1.800959822
H	-1.070299859	0.482449190	-0.302945519
H	0.052923317	-0.682128032	1.776935683
H	1.363072761	-1.801954548	1.346886673
H	-4.589700175	-2.558609925	0.748019585
H	-6.937470606	-1.709109881	0.753874767
H	-5.604426443	1.984980194	-1.023682731
H	-3.272128207	1.148457138	-1.029020954
Cl	-8.044115756	0.777847624	-0.131658710

HF= -2018.8876016 hartree

Imaginary Freq 0

The converged geometry of 6

C	4.637878202	0.624179858	-0.388524645
C	4.169628294	-0.683652327	-0.123184929
C	2.863603148	-0.980799503	0.237025516
C	1.990898910	0.114291259	0.330270130
C	2.428986730	1.441288492	0.068527743
C	3.765930438	1.695972575	-0.292345493
N	0.662183416	0.199263546	0.665496870
N	0.224854192	1.478809842	0.623821457
C	1.251229509	2.223484995	0.276767336
N	5.126425476	-1.809484703	-0.235108921
O	4.705290123	-2.932404292	-0.008651413
O	6.274861685	-1.543107126	-0.546779843
Cl	1.122886335	3.933757420	0.117868868
C	-1.066649383	-1.363663407	-0.255743473
O	-1.809926900	-2.526507768	0.171999055

N	-3.153469945	-2.202512367	0.310255211
C	-3.375270261	-1.031473643	-0.172938065
C	-2.135781327	-0.382196955	-0.751532825
C	-0.262241766	-0.877781440	0.963991452
H	5.683010958	0.755546264	-0.664591500
H	2.572102416	-2.012781500	0.426678830
H	4.101167427	2.715759973	-0.490624786
H	-0.376626207	-1.686477568	-1.050466730
H	-2.202521151	-0.342150699	-1.851970498
H	-1.973116056	0.643858145	-0.389154914
H	-0.948753529	-0.531234373	1.749947961
H	0.309381605	-1.725409456	1.367370500
C	-4.704601283	-0.433076046	-0.186591787
C	-5.067023297	0.872741159	-0.681271215
C	-5.877111056	-0.991468483	0.266327140
H	-4.407807354	1.619127555	-1.118910076
C	-6.411417461	0.990691535	-0.487708483
H	-6.104709714	-1.952112942	0.719502006
H	-7.130197225	1.778858782	-0.694846098
O	-6.905138300	-0.136808676	0.086386178

HF= -1557.1928519 hartree

Imaginary Freq 0

The converged geometry of 7

C	5.209101314	0.729525888	-0.390567520
C	4.781423807	-0.595572827	-0.142688685
C	3.486770161	-0.936817174	0.219550628
C	2.582395754	0.130236391	0.333935862
C	2.979566865	1.473610554	0.090604760
C	4.306299294	1.773291123	-0.273380672
N	1.253742497	0.170500382	0.677273535
N	0.778594663	1.436867480	0.657914654
C	1.780391665	2.217096301	0.317269298
N	5.770685997	-1.690355353	-0.276228703

O	5.385168994	-2.828568921	-0.062826264
O	6.908624996	-1.385354599	-0.591252264
Cl	1.600302514	3.925089158	0.186161036
C	-0.432949297	-1.426974364	-0.263140374
O	-1.129401142	-2.624440845	0.143885975
N	-2.482973562	-2.354107059	0.290631634
C	-2.756304576	-1.181688421	-0.164482141
C	-1.540383007	-0.474905443	-0.730064463
C	-4.121497618	-0.643286491	-0.157706248
C	0.362829257	-0.938411379	0.961251743
C	-5.194352943	-1.401861707	0.351853174
C	-6.489200325	-0.896197444	0.354905652
C	-6.750979072	0.387884698	-0.154315393
C	-5.694022501	1.154158959	-0.664640089
C	-4.399176135	0.640929149	-0.663073423
H	6.248307888	0.896161110	-0.669979308
H	3.226989758	-1.979730391	0.394952896
H	4.610052702	2.805501994	-0.457687910
H	0.263323571	-1.706541445	-1.068982166
H	-1.611138376	-0.408331065	-1.828780776
H	-1.409521045	0.546215497	-0.342735762
H	-0.329416971	-0.625864441	1.756373204
H	0.961518247	-1.775527828	1.346942549
H	-4.994012109	-2.399654155	0.745994212
H	-7.310807114	-1.499416332	0.754582600
H	-5.907681467	2.150387154	-1.056342947
H	-3.591699168	1.257727276	-1.063546420
O	-7.993719826	0.926493227	-0.174554745
H	-8.626265976	0.301080978	0.203440075

HF= -1634.5876467 hartree

Imaginary Freq 0

The converged geometry of 8

C	5.779431180	0.850297614	-0.404150263

C	5.403527846	-0.492944443	-0.172660159
C	4.126412949	-0.887704016	0.197280604
C	3.184723926	0.143324599	0.336758360
C	3.529380080	1.503691788	0.110521992
C	4.840043422	1.858198216	-0.261711343
N	1.858090719	0.130069865	0.692272392
N	1.336318162	1.379329922	0.696671469
C	2.305816672	2.200183431	0.359153678
N	6.431212183	-1.548855404	-0.333813876
O	6.089129096	-2.703234972	-0.134430919
O	7.553031649	-1.196827341	-0.655881084
Cl	2.062711046	3.901299217	0.255090836
C	0.200376760	-1.490169182	-0.246945839
O	-0.474515838	-2.709350753	0.155658922
N	-1.823546896	-2.481655170	0.284831431
C	-2.128233953	-1.308168123	-0.147824743
C	-0.931423492	-0.550645377	-0.683623799
C	-3.515843175	-0.826304983	-0.138048075
C	1.008848913	-1.010592854	0.971520396
C	-4.557626937	-1.646724350	0.346188955
C	-5.871054463	-1.193975954	0.351729493
C	-6.146660029	0.089349404	-0.130635459
C	-5.141714978	0.924325823	-0.614984943
C	-3.826775878	0.460593865	-0.615989750
H	6.808844551	1.059503469	-0.691234622
H	3.908415963	-1.942396772	0.359045702
H	5.103528156	2.903637508	-0.432785261
H	0.884461022	-1.751664797	-1.068412602
H	-0.999508824	-0.444618135	-1.779217643
H	-0.826215578	0.458214356	-0.257725879
H	0.325615043	-0.738034593	1.789128541
H	1.640102930	-1.838412082	1.323864473
H	-4.315646882	-2.644243425	0.715452551
H	-6.689473647	-1.811585738	0.720643648

H	-5.401398489	1.917457781	-0.980126818
H	-3.036731761	1.112675289	-0.992667197
N	-7.542088158	0.574937217	-0.126496801
O	-8.399056104	-0.179102201	0.304939705
O	-7.749008210	1.699038104	-0.554816130

HF= -1763.7674009 hartree

Imaginary Freq 0

The converged geometry of 9

C	-5.846501327	0.878191637	0.403643364
C	-5.476910389	-0.468335575	0.177906017
C	-4.202641414	-0.869816783	-0.194872660
C	-3.257130863	0.156651290	-0.344244154
C	-3.595983360	1.520111999	-0.124275379
C	-4.903930647	1.881361246	0.251642828
N	-1.933154892	0.135033807	-0.706170862
N	-1.406868189	1.380659226	-0.719974122
C	-2.371068751	2.208412439	-0.382932160
N	-6.508288648	-1.517808437	0.348501176
O	-6.174807232	-2.675132972	0.150712642
O	-7.627134668	-1.159518572	0.676265637
Cl	-2.119291471	3.910463419	-0.291359076
C	-0.320230093	-1.526409785	0.256759933
O	0.325626646	-2.754155386	-0.134971019
N	1.692622390	-2.538189457	-0.290134331
C	2.011737989	-1.372472309	0.155331316
C	0.823388925	-0.615612722	0.716907143
C	3.391046257	-0.881027895	0.144546747
C	-1.091772401	-1.016362463	-0.974957862
C	4.447169341	-1.676200824	-0.346318028
C	5.756371911	-1.220339871	-0.355855373
C	6.090946026	0.073329504	0.130480603
C	5.027899419	0.869921227	0.624520707
C	3.718388133	0.397907328	0.626878059

H	-6.873811165	1.092916535	0.693938702
H	-3.987502387	-1.925840673	-0.351348249
H	-5.162672345	2.928783074	0.418029128
H	-1.030602480	-1.767420806	1.063146648
H	0.897391469	-0.544925744	1.815137036
H	0.733638685	0.407423608	0.323068395
H	-0.384862756	-0.742605848	-1.771227956
H	-1.726355905	-1.830149387	-1.353334264
H	4.219270422	-2.674551687	-0.724750757
H	6.531669604	-1.878403695	-0.746262912
H	5.219565524	1.870314427	1.009948507
H	2.934802333	1.052187874	1.016371940
N	7.392515626	0.530586543	0.120380623
C	7.701009029	1.851784392	0.628386500
H	7.429960367	1.963375512	1.694829594
H	8.779063102	2.035371225	0.536921986
H	7.175991589	2.647377112	0.067785727
C	8.457060969	-0.311593365	-0.387642154
H	8.559491534	-1.250119961	0.188146328
H	8.301692041	-0.587090127	-1.447014872
H	9.410870733	0.226967255	-0.319988834

HF= -1693.2951671 hartree

Imaginary Freq 0

The converged geometry of 10

C	2.637830426	-3.064281364	-0.055966134
C	1.775071201	-2.095528142	0.507442739
C	2.134934892	-0.774374157	0.726332219
C	3.436092449	-0.414932100	0.343806923
C	4.328763990	-1.366845911	-0.222281916
C	3.923481465	-2.700407880	-0.422294049
N	4.116253879	0.775750881	0.395411744
N	5.371295765	0.647390054	-0.085005943
C	5.515164983	-0.605325061	-0.454488502

N	0.402935753	-2.501279470	0.880960621
O	-0.339111066	-1.645464400	1.344414717
O	0.089738480	-3.666279922	0.705022680
Cl	6.985361941	-1.193401876	-1.134668858
C	2.336852214	2.492786625	0.401692507
N	0.252414874	2.991029093	0.109317260
C	3.679077571	2.069332715	0.917379694
H	2.266758872	-4.079152632	-0.190573980
H	1.420544463	-0.082084426	1.161577982
H	4.611433564	-3.426417406	-0.859755197
H	4.457545534	2.778165714	0.605510316
H	3.664768031	2.040576249	2.020022926
C	1.134345817	2.628121384	1.073789736
H	0.848930654	2.498226229	2.114744510
N	2.117573132	2.780326302	-0.911929597
N	0.865856389	3.072832879	-1.082565717
C	-1.196058499	3.138060087	0.203627712
C	-1.937029999	1.834915761	-0.111821035
H	-1.480962461	3.933449540	-0.500619956
H	-1.428248051	3.489857334	1.220801737
C	-3.454021138	1.963424921	0.046083307
H	-1.685349029	1.534812511	-1.143070579
H	-1.559243260	1.031748609	0.544857427
H	-3.690505048	2.276142740	1.080479097
H	-3.826892594	2.774777450	-0.606333091
C	-4.205779207	0.667156814	-0.272584220
C	-5.722440907	0.775165150	-0.090635258
H	-3.815226977	-0.145421325	0.366904795
H	-3.981242797	0.363399380	-1.311534230
C	-6.477068514	-0.519483820	-0.406890338
H	-5.943657106	1.080880298	0.949116497
H	-6.110431285	1.589957681	-0.730224434
H	-6.259436221	-0.823563770	-1.447663458
H	-6.084860805	-1.334193680	0.229707018

C	-7.992999563	-0.414576654	-0.216787857
C	-8.749959782	-1.708793719	-0.531132358
H	-8.385541447	0.400696679	-0.853358333
H	-8.209934453	-0.110035075	0.824417206
C	-10.262628189	-1.595781520	-0.336182910
H	-8.533168723	-2.011797225	-1.571755047
H	-8.355709926	-2.522551671	0.104560560
H	-10.773942055	-2.543402985	-0.569603242
H	-10.513945524	-1.330120306	0.704570588
H	-10.693939503	-0.816310449	-0.986767818

HF= -1677.0902391 hartree

Imaginary Freq 0

The converged geometry of 11

C	2.454233883	2.711503986	0.412600733
C	1.361847826	2.388116408	-0.424944151
C	1.114829082	1.113952739	-0.916253071
C	2.029448446	0.121610190	-0.535010338
C	3.139913229	0.414202756	0.303816496
C	3.351372621	1.721456428	0.780159774
N	2.109575893	-1.213596005	-0.835977369
N	3.179889014	-1.790422797	-0.248940894
C	3.799459601	-0.848499788	0.425177746
N	0.422548924	3.466234226	-0.805187603
O	-0.498239488	3.177552331	-1.554315947
O	0.624852633	4.579187026	-0.348473650
Cl	5.238235167	-1.162342833	1.320228782
C	-0.140899388	-2.252048226	-0.974059160
N	-2.223120450	-2.328241161	-0.395238351
C	1.190400769	-2.021250294	-1.628791512
H	2.563757063	3.741034696	0.749903282
H	0.250641023	0.934681917	-1.552276869
H	4.204985214	1.944531589	1.423144046
H	1.707842084	-2.977281640	-1.784002639

H	1.048972889	-1.542943711	-2.610878662
C	-1.399117552	-1.801911473	-1.335606313
H	-1.757949676	-1.178723245	-2.150822899
N	-0.272509142	-3.008800733	0.151974483
N	-1.519909102	-3.049400826	0.496924993
C	-3.663524115	-2.155285181	-0.224206556
H	-3.966250729	-2.960396515	0.460974341
H	-4.153142835	-2.333042550	-1.193059557
C	-4.045930112	-0.795086906	0.326132595
C	-4.908633996	0.047036584	-0.386978489
C	-3.544515665	-0.368520809	1.566659403
C	-5.269284690	1.296615787	0.128511505
H	-5.305416479	-0.274804606	-1.354478964
C	-3.900880702	0.879711381	2.079205479
H	-2.868570281	-1.019874506	2.127739892
C	-4.764684253	1.715317003	1.361432904
H	-5.941780961	1.945032905	-0.438504530
H	-3.504211547	1.202784363	3.044999812
H	-5.041314676	2.692982094	1.763721180

HF= -1593.690567 hartree

Imaginary Freq 0

The converged geometry of 12

C	2.217376601	-3.016210241	-0.071260810
C	1.337200449	-2.054343203	0.476826041
C	1.685851885	-0.732712096	0.710510004
C	2.993229719	-0.364298698	0.358745247
C	3.903431913	-1.309729508	-0.190179485
C	3.509463372	-2.644351440	-0.405464792
N	3.666010280	0.829889105	0.429583846
N	4.932291838	0.709081802	-0.022406177
C	5.091044658	-0.541707896	-0.392310064
N	-0.041424370	-2.468170104	0.815856198
O	-0.797065865	-1.619152400	1.270556226

O	-0.346687645	-3.632317771	0.622218331
Cl	6.579304923	-1.120176757	-1.040606406
C	1.871303248	2.529431314	0.406005803
N	-0.217951409	2.983614934	0.077683808
C	3.210659337	2.121908856	0.941354948
H	1.854468315	-4.032098419	-0.219762234
H	0.959047048	-0.046317990	1.133790953
H	4.211064317	-3.364980843	-0.829954602
H	3.986186328	2.836684753	0.635964985
H	3.182972778	2.097359127	2.043840088
C	0.654250409	2.636290436	1.056631486
H	0.352679201	2.494565021	2.091487043
N	1.669684716	2.818304184	-0.910217397
N	0.415081972	3.083546062	-1.102589086
C	-1.672801892	3.075318858	0.143275928
C	-2.349029371	1.737264503	-0.171636562
H	-1.975377416	3.851324813	-0.574954965
H	-1.938597491	3.427213315	1.152250153
C	-3.871919402	1.784864658	-0.027289896
H	-2.073082576	1.444101804	-1.198701427
H	-1.937736604	0.957160345	0.492430175
H	-4.133828940	2.098892450	1.000604438
H	-4.285622047	2.563249862	-0.695032427
C	-4.542215095	0.440957276	-0.330116670
C	-6.062740614	0.453546768	-0.148900600
H	-4.099703490	-0.337336404	0.318097460
H	-4.299043162	0.139414174	-1.365541076
C	-6.729530883	-0.893762090	-0.440393268
H	-6.303812019	0.763114160	0.885329561
H	-6.503571827	1.228938293	-0.803132057
H	-6.490178880	-1.203883449	-1.474777617
H	-6.285680766	-1.668140598	0.212667602
C	-8.250204048	-0.887260576	-0.254675182
C	-8.907287407	-2.237981456	-0.542003902

H	-8.692566012	-0.114706743	-0.910101470
H	-8.487418280	-0.573362718	0.778394888
H	-8.719124783	-2.562661740	-1.579210758
H	-8.514521151	-3.024528670	0.124067424
H	-9.999056855	-2.196449006	-0.399731315

HF= -1637.8048048 hartree

Imaginary Freq 0

The converged geometry of 13

C	2.335524656	2.676720647	0.240703735
C	1.126282034	2.228400972	-0.340196109
C	0.874727405	0.907653470	-0.685699915
C	1.911222850	-0.000899681	-0.427204306
C	3.141305674	0.418174691	0.151333996
C	3.351773902	1.768603851	0.488138604
N	2.019629440	-1.351700615	-0.636863845
N	3.223571881	-1.817995884	-0.242013425
C	3.896941818	-0.792365750	0.227630562
N	0.058865433	3.217923888	-0.588796305
O	-0.953728880	2.829077883	-1.158314694
O	0.243976380	4.362675462	-0.215063131
Cl	5.497100036	-0.956725224	0.844700470
C	-0.275032370	-2.285158381	-0.561477731
N	-2.369593701	-2.265236932	-0.030536836
C	1.061000263	-2.265778444	-1.244022998
H	2.437878289	3.733563989	0.482247847
H	-0.084435883	0.626406668	-1.114205632
H	4.296202126	2.086878793	0.933645995
H	1.539964549	-3.252985632	-1.185315090
H	0.930266335	-2.017402106	-2.310715985
C	-1.538151196	-2.092052321	-1.089491187
H	-1.894697782	-1.854713890	-2.088363447
N	-0.410830853	-2.551617541	0.769526169
N	-1.664456974	-2.532959423	1.085039621

C	-3.808193211	-2.143057282	0.038551514
H	-4.135667349	-2.669640229	0.945881239
H	-4.274522041	-2.645849774	-0.822014360
C	-4.370842752	-0.720744461	0.105367642
O	-5.557906630	-0.526404916	0.171432727
O	-3.424684827	0.214250893	0.073605411
C	-3.842712739	1.602470114	0.166991729
C	-3.995370845	2.031167764	1.615036063
H	-4.786461156	1.717340228	-0.385384933
H	-3.046047789	2.162343183	-0.339029194
H	-4.232144792	3.106244183	1.656207148
H	-4.810946727	1.482962994	2.109911079
H	-3.062486654	1.864680267	2.175487576

HF= -1629.7963357 hartree

Imaginary Freq 0

The converged geometry of 14

C	4.107172795	-0.837794177	-1.157897353
C	3.314498640	-1.625827934	-0.291794932
C	2.324496460	-1.113952141	0.534172201
C	2.145070838	0.276353704	0.476207346
C	2.919569564	1.097511186	-0.386683743
C	3.911379393	0.532546462	-1.210306068
N	1.294338076	1.118195994	1.149501506
N	1.454154786	2.403957572	0.763132348
C	2.411025751	2.411205925	-0.136945897
N	3.548670464	-3.088795839	-0.263508704
O	2.879656994	-3.752592113	0.511118418
O	4.398826015	-3.537015716	-1.014789447
Cl	2.950626913	3.868106013	-0.881123470
C	-1.002893500	0.261134569	1.484128031
O	-1.046957063	-1.060460121	1.223418418
N	-2.242882521	-1.374744596	0.620584401
C	-2.918098694	-0.245293272	0.518343738

C	-2.159975226	0.844874849	1.063404616
C	-4.261932348	-0.220431990	-0.090906892
C	0.255147242	0.779859879	2.110270840
C	-4.837522353	-1.405987591	-0.585112290
C	-6.105436486	-1.386195055	-1.164076662
C	-6.819770526	-0.186208531	-1.260188014
C	-6.256265867	0.995594293	-0.772028849
C	-4.986016516	0.979242970	-0.191290044
H	4.857100080	-1.335209515	-1.771061958
H	1.729000548	-1.778522140	1.158043967
H	4.507652094	1.163575467	-1.871991030
H	-2.427527381	1.895399535	1.126736401
H	0.042745365	1.697511247	2.672315013
H	0.651084579	0.027123539	2.809529996
H	-4.275460564	-2.338226593	-0.508698219
H	-6.540675566	-2.313790743	-1.544294838
H	-7.813433476	-0.173291724	-1.715031978
H	-6.807002062	1.936777709	-0.843123939
H	-4.559009175	1.911303746	0.184953412

HF= -1558.2224601 hartree

Imaginary Freq 0

The converged geometry of 15

C	4.710330209	-0.826463884	-1.359370398
C	4.000021874	-1.618771075	-0.428277857
C	3.075026382	-1.113698591	0.473737211
C	2.875091099	0.274039944	0.424540075
C	3.566785042	1.099261937	-0.502303263
C	4.494836527	0.541344907	-1.401508793
N	2.071381901	1.109793945	1.160500603
N	2.182217679	2.395007370	0.754987617
C	3.063472077	2.408394840	-0.219073047
N	4.252355605	-3.079020062	-0.412937383
O	3.654935108	-3.746097129	0.415568245

O	5.044667120	-3.521477638	-1.227949345
Cl	3.522058828	3.866724183	-1.012607728
C	-0.195431735	0.257318030	1.664033515
O	-0.267458036	-1.061065785	1.393964430
N	-1.507128984	-1.362916002	0.881769992
C	-2.181302403	-0.228784244	0.843080917
C	-1.377807230	0.851853496	1.338884931
C	-3.566779877	-0.193261652	0.340319837
C	1.110203624	0.763816918	2.196117604
C	-4.181833196	-1.368412905	-0.130517334
C	-5.489680548	-1.348104770	-0.607814729
C	-6.201284986	-0.142700256	-0.619082966
C	-5.611037242	1.036701232	-0.157557439
C	-4.299714762	1.004440332	0.318392968
H	5.414103188	-1.318588793	-2.028929677
H	2.541522390	-1.782145470	1.147578079
H	5.027652198	1.175606598	-2.112386443
H	-1.632204695	1.903443188	1.432327478
H	0.945799884	1.674636174	2.784867066
H	1.556533914	0.000997243	2.852511826
H	-3.619018430	-2.303218946	-0.118413893
H	-5.963832782	-2.260626454	-0.972614316
H	-6.176270036	1.969736566	-0.172357097
H	-3.849612517	1.932469968	0.676678205
Cl	-7.841998782	-0.112905829	-1.215261738

HF= -2017.6883717 hartree

Imaginary Freq 0

The chosen basis functions in this study

```
H     0
S   3   1.00
     13.0107010              0.19682158E-01
      1.9622572              0.13796524
      0.44453796             0.47831935
S   1   1.00
      0.12194962             1.0000000
P   1   1.00
      0.8000000              1.0000000
****
C     0
S   5   1.00
   1238.4016938              0.54568832082E-02
    186.29004992             0.40638409211E-01
     42.251176346            0.18025593888
     11.676557932            0.46315121755
      3.5930506482           0.44087173314
S   1   1.00
      0.40245147363          1.0000000
S   1   1.00
      0.13090182668          1.0000000
P   3   1.00
      9.4680970621           0.38387871728E-01
      2.0103545142           0.21117025112
      0.54771004707          0.51328172114
P   1   1.00
      0.15268613795          1.0000000
D   1   1.00
      0.8000000              1.0000000
****
N     0
S   5   1.00
```

1712.8415853 -0.53934125305E-02
257.64812677 -0.40221581118E-01
58.458245853 -0.17931144990
16.198367905 -0.46376317823
5.0052600809 -0.44171422662
S 1 1.00
0.58731856571 1.0000000
S 1 1.00
0.18764592253 1.0000000
P 3 1.00
13.571470233 -0.40072398852E-01
2.9257372874 -0.21807045028
0.79927750754 -0.51294466049
P 1 1.00
0.21954348034 1.0000000
D 1 1.00
1.0000000 1.0000000
****
O 0
S 5 1.00
2266.1767785 -0.53431809926E-02
340.87010191 -0.39890039230E-01
77.363135167 -0.17853911985
21.479644940 -0.46427684959
6.6589433124 -0.44309745172
S 1 1.00
0.80975975668 1.0000000
S 1 1.00
0.25530772234 1.0000000
P 3 1.00
17.721504317 0.43394573193E-01
3.8635505440 0.23094120765
1.0480920883 0.51375311064
P 1 1.00

0.27641544411 1.0000000
D 1 1.00
1.2000000 1.0000000
****
Cl 0
S 5 1.00
10449.8275660 0.19708362484E-02
1571.7365221 0.14754727977E-01
357.12065523 0.66679112875E-01
100.25185935 0.17228924084
30.812727554 0.15883786100
S 3 1.00
51.923789434 -0.10009298909
5.7045760975 0.60841752753
2.3508376809 0.54352153355
S 1 1.00
0.44605124672 1.0000000
S 1 1.00
0.16848856190 1.0000000
P 5 1.00
307.66790569 -0.87801484118E-02
72.102015515 -0.63563355471E-01
22.532680262 -0.24016428276
7.8991765444 -0.47798866557
2.8767268321 -0.38515850005
P 1 1.00
0.77459363955 1.0000000
P 1 1.00
0.21037699698 1.0000000
D 1 1.00
0.6500000 1.0000000
****
